# A Systematic Review of Built Environment Interventions to Improve Patient and Staff Outcomes in Emergency Department Mental and Behavioral Healthcare

**DOI:** 10.1007/s10488-026-01502-0

**Published:** 2026-04-07

**Authors:** Monica Gripko, Sara Kennedy, Anjali Joseph, RIPCHD.PED Study Group

**Affiliations:** School of Architecture, Clemson University, Clemson, USA

**Keywords:** Emergency Department Design, Healthcare Design, Physical Environment, Patient Safety, Staff Safety, Outcomes

## Abstract

The built environment of Emergency Departments (EDs) provides the context and setting for care delivery, influences workflows, and shapes user interactions, but its noisy, sterile, stimulating nature can be counter-therapeutic to patients in psychiatric crises. As ED Mental and Behavioral Health (MBH) presentation rates rise, healthcare organizations and designers must develop strategies to address challenges and improve care. This systematic review analyzes emergency department-based built environment interventions implemented to improve the safety and experiences of patients seeking emergency MBH care and ED staff involved in their care. Our team searched six databases in March 2023 using PRISMA methods and the SEIPS 3.0 framework to identify and analyze nineteen studies of ED-based built environment interventions for MBH care. Interventions occurred at three scales – unit, room, and element – and impacted patient and staff outcomes. For example, MBH units located outside of the main ED contributed to decreases in length of stay, boarding, and inpatient psychiatric admissions. Room and element-scale interventions helped improve patient experiences and increase safety. This review summarizes existing knowledge surrounding built environment interventions for emergency MBH care and identifies opportunities for design and clinical practice applications. Future research is necessary to capture patient and staff voices, examine various types of EDs, and compare interventions across hospitals. This review shows how physical interventions in the built environment of EDs – from individual elements to entire units – can improve processes, safety, and experiences for MBH patients and staff, especially when incorporated into broader, systems-based changes.

**Supplementary Information:**

The online version contains supplementary material available at 10.1007/s10488-026-01502-0.

## Introduction

Millions of Americans seek care for mental and behavioral health (MBH) conditions in emergency departments (EDs) every year. From 2007 to 2016, an average of over 8% of all ED visits were for psychiatric or substance use-related concerns, increasing from 6.6% in 2007 to 10.9% in 2016 (Theriault et al., 2020). Moreover, these numbers continue to rise for adults and children (Bommersbach et al., 2023; Brathwaite et al., 2022; Weiss et al., 2016). EDs deliver services to people with emergency health needs and those seeking after-hours care and increasingly provide a safety net for people who have difficulty accessing alternative care sources (Moore et al., 2017; ). ED built environments provide the context and setting for care delivery, influence workflows, and shape interactions between patients, families, clinicians, and staff. However, their noisy, sterile, stimulating nature can be counter-therapeutic to patients experiencing psychiatric crises (Chun et al., 2015; Innes et al., 2014).

Studies show thoughtful, evidence-based environmental design can improve health outcomes and help make healthcare environments more comfortable, therapeutic, and safe for patients and staff (Gripko et al., 2023; Rowe & Knox, 2023; Ulrich et al., 2008). Yet, our team found no comprehensive reviews exploring built environment interventions implemented by hospitals or health systems to improve the care, safety, and experiences of patients and staff involved in ED-based MBH care. This systematic review summarizes and analyzes built environment interventions implemented by hospitals and health systems, identifies knowledge gaps, and highlights opportunities for future research, design, and clinical practice to improve the safety and experiences of patients and staff in the emergency MBH care process.

### Theoretical Framework

**Fig. 1:**
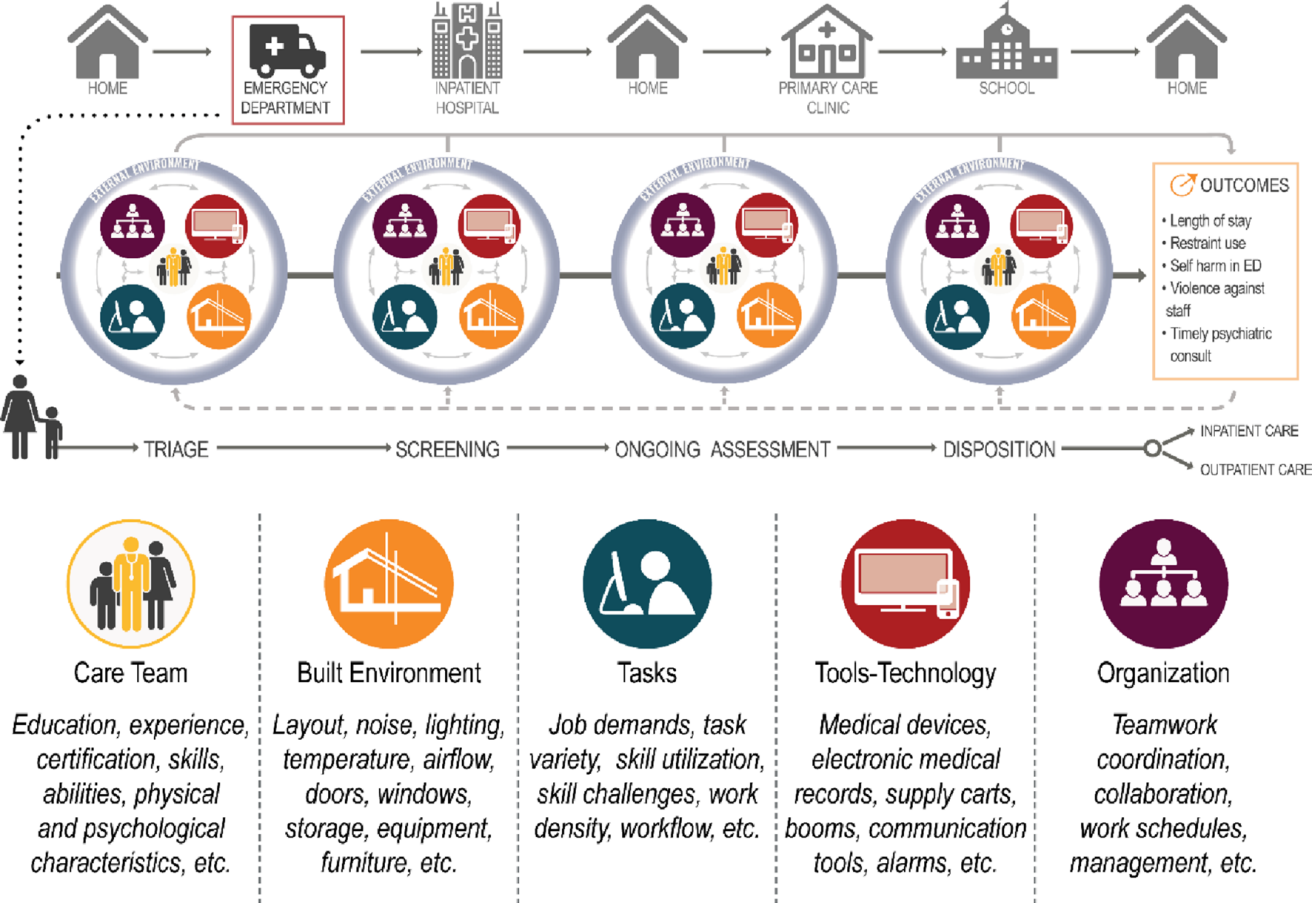
The SEIPS 3.0 Framework

The Systems Engineering Initiative for Patient Safety (SEIPS) 3.0 model (Carayon et al., 2020) provides the theoretical framework for this review (Fig. 1). SEIPS 3.0 is a sociotechnical systems approach that conceptualizes the patient journey in terms of dynamic interactions within work systems and across work systems and the external environment. The built environment is one work system component that dynamically interacts with other work system components (including care teams, tasks, organizational conditions, and tools and technologies) over time to produce the outcomes experienced by patients, care partners, clinicians, and healthcare organizations. The review presented in this paper specifically focuses on the built environment, though many of the included studies also involve other components of ED work systems.

#### Scope and Objective

The review presented in this paper is part of a larger systematic literature review project that aims to identify and assess ED-based interventions to improve the safety and care experiences for MBH patients and staff. Although the larger review included interventions for all SEIPS 3.0 work systems, this paper focuses on built environment interventions, meaning changes to the ED’s physical environment to make patients or staff safer, improve care quality or timeliness, or enhance the care process. Additional review papers (currently under peer review) focus on the other SEIPS 3.0 work systems.

## Methods

Our team developed a review protocol using the Preferred Reporting Items for Systematic reviews and Meta-Analyses (PRISMA) 2020 Statement (Page et al., 2021) and registered it with the International Prospective Register of Systematic Reviews (PROSPERO; # CRD42023395596).

### Search Strategy

We developed a comprehensive list of keywords with a research librarian using a Population, Intervention, Outcome (PIO) framework. Full search strings are available as supplemental material. Population keywords included MBH conditions, care providers, and patients. As mentioned above, this paper represents a subset of a broader review, so intervention keywords included terms related to all SEIPS 3.0 categories. Patient and staff outcome keywords included terms like length of stay, anxiety, aggression, injury, or satisfaction. Experts from this study’s technical and clinical advisory committees reviewed the keyword lists for relevance and comprehensiveness. We ran searches in March 2023, with the keywords tailored to the search interfaces of six databases to search (PubMed, CINAHL, Web of Science, Medline, PsycINFO, and Engineering Village). Additionally, we hand-searched reference lists from related literature reviews.

### Study Selection

**Fig. 2:**
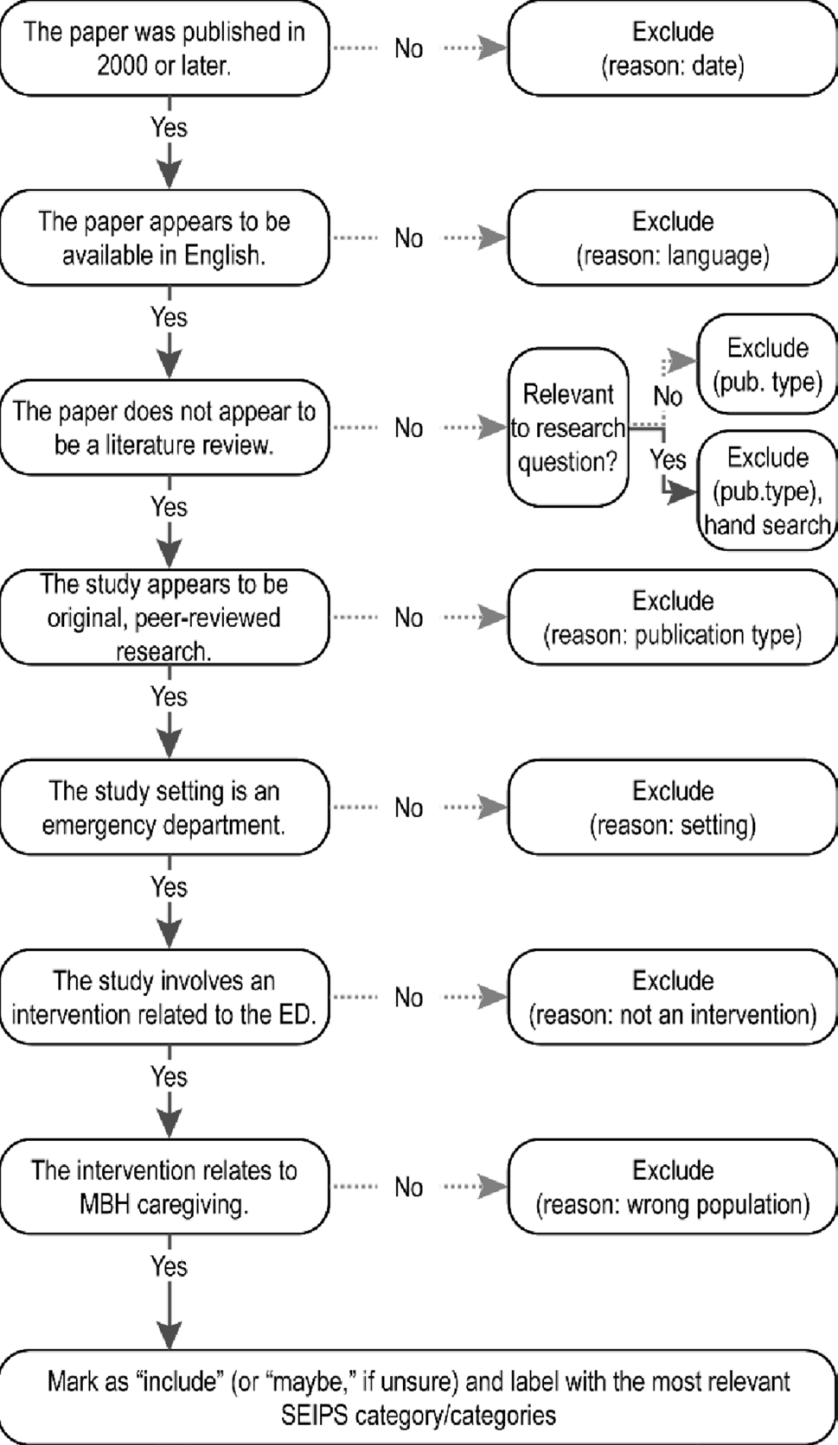
Decision Flowchart

Articles were eligible for inclusion if they presented original research published in English in a peer-reviewed journal between 2000 and March 2023 and were available in full-text through open access or our institution’s libraries. We selected 2000 as the cutoff date for this paper because it coincides with a significant turning point in patient safety and healthcare quality improvements. In November 1999, the United States Institute of Medicine published its landmark report, *To Err is Human*, and a second report, *Crossing the Quality Chasm*, in 2001 (Institute of Medicine, 1999, 2001), which ushered in a push for improved safety and quality in healthcare in the United States, specifically, but also around the world. While not explicitly focused on mental health, these reports spurred new research and a focus on health system challenges.

To be included in this review, studies needed to address an ED-based intervention related to the MBH caregiving process or environment for patients (adults or children) or staff. We excluded studies focused only on pharmaceutical interventions (excluded as “pharmacologic study/outcome”) or medical case reports (excluded as “wrong publication type”). The intervention needed to involve the built environment for inclusion in the current sub-review.

After running the searches and collecting all database returns, one team member uploaded the search results into Rayyan.ai (Ouzzani et al., 2023) and removed all duplicate titles and those not meeting the preliminary eligibility criteria (publication period, full-text, language). Before starting the title and abstract review, the team met to discuss inclusion criteria and practice using the decision flowchart (Fig. 2).

**Fig. 3:**
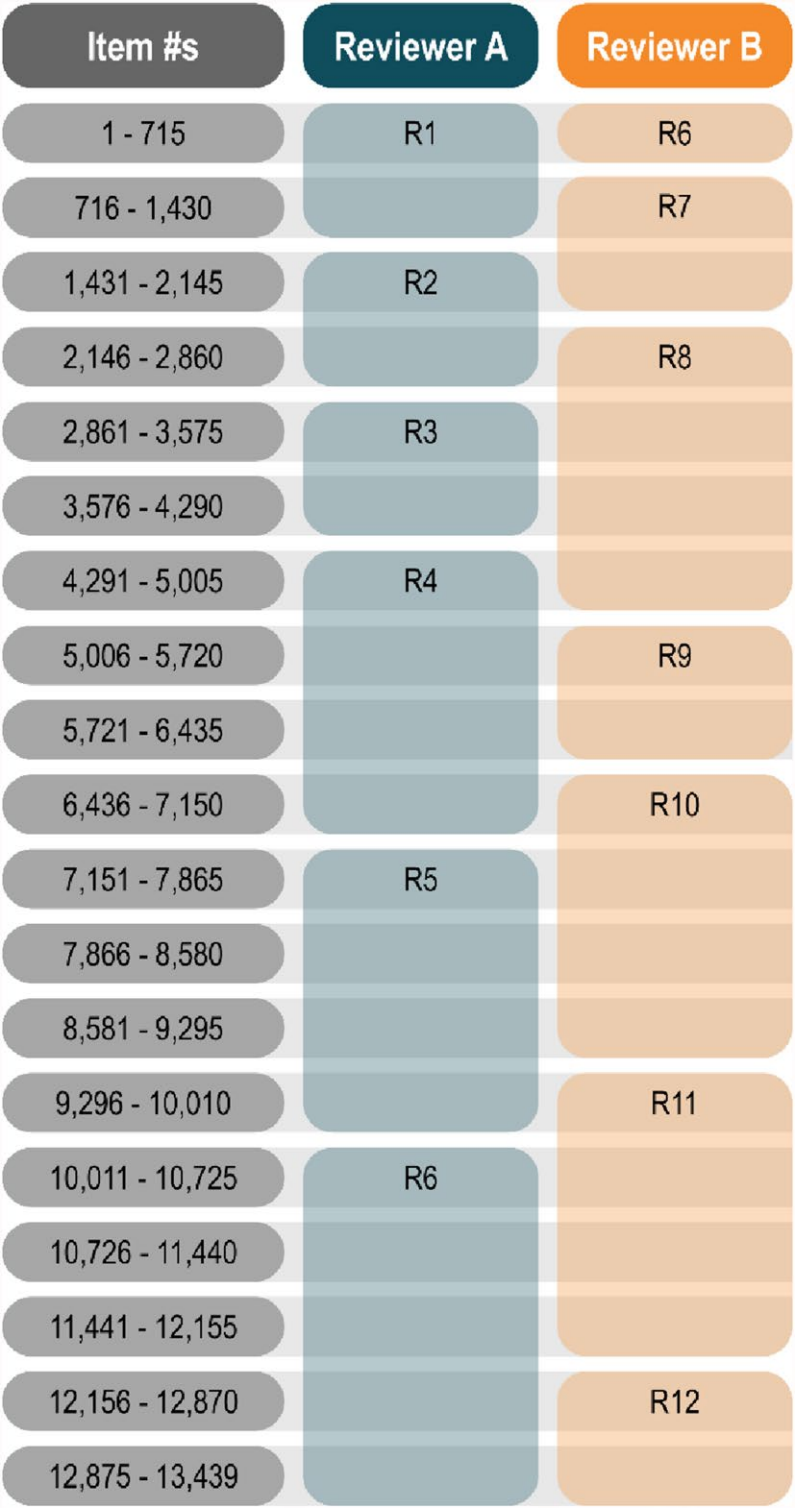
Reviewer Assignments

Next, the team coordinator split up the titles between twelve team members, so each title was reviewed by two independent reviewers (Fig. 3). Titles were staggered between reviewers to support consistency and reduce bias. Each reviewer used the decision flowchart to evaluate titles and abstracts in Rayyan.ai. They marked items as “include” if they met all criteria, “exclude” if they failed to meet any requirements, or “maybe” if they felt unsure based on the title and abstract. They also assigned labels with the most relevant SEIPS categories for each included or “maybe” item. After independently reviewing their assignments, pairs met to discuss all discrepancies and “maybe” items. The team coordinator acted as a tie-breaker for any non-consensus.

### Data Extraction

The included papers labeled “built environment” moved forward to a full review. Two reviewers (A1 and A2) split the included papers evenly. Each reviewer extracted data from their assigned papers using a spreadsheet template developed by the research team before verifying the other’s extracted data. The spreadsheet collected citation information, study setting, design, methods, built environment elements, outcome measures, and findings.

Additionally, the two reviewers independently evaluated each paper’s research design, methodological quality, and risk of bias. They assigned each a level of evidence, ranging from 1 (high) to 6 (low), using the Levels of Evidence for Healthcare Design model (Table 1; Marquardt et al., 2014; Stichler, 2010). They also independently scored each paper for quality using the Mixed Methods Appraisal Tool, version 2018 (Hong et al., 2018). They discussed any discrepancies until reaching a consensus and consulted a third researcher (A3) as a tie-breaker where they could not agree.

**Table 1:** Levels of Evidence for Healthcare Design Model

Level	Description of Quality	Included?
1	Systematic reviews of multiple randomized controlled trials or non-randomized studies; meta-analysis of multiple experimental or quasi-experimental studies; meta-synthesis of multiple qualitative studies leading to an integrative interpretation	No
2	Well-designed experimental (randomized) or quasi-experimental (non-randomized) studies with a low attrition rate, intention to treat analysis, blinding, masked randomization, and consistent results compared to other, similar studies	Yes
3a	Observational studies with a cohort design; experimental or quasi-experimental studies that did not fulfill the criteria of Level 2	Yes
3b	Cross-sectional studies or case-control studies; qualitative research that, based on a literature review or a theoretical framework, reports a clear method and considers a diversity of views	Yes
4	Professional standards or guidelines with studies to support recommendations	No
5	Qualitative research that did not meet the criteria of Level 3b	Yes
6	Recommendations from manufacturers or consultants who may have a financial interest or bias	No

### Data Synthesis

The team synthesized and described the data using a hybrid inductive-deductive thematic coding approach that integrated data-driven and theory-driven codes (Braun & Clarke, 2006; Fereday & Muir-Cochrane, 2006; Proudfoot, 2023). Such an approach enables the use of an overarching framework to guide deductive thematic analysis, while also allowing themes to emerge inductively, directly from the data (Fereday & Muir-Cochrane, 2006). In this review, the SEIPS 3.0 Framework served as the overarching organizing framework, guiding the organization of codes. Specific intervention types, scales, and outcome themes were derived inductively from the data. One reviewer coded the interventions by type, scale, and outcomes, grouped these codes into categories, and grouped categories under broader themes in a spreadsheet. The other authors reviewed and verified the codes, categories, and themes.

## Results

**Fig. 4:**
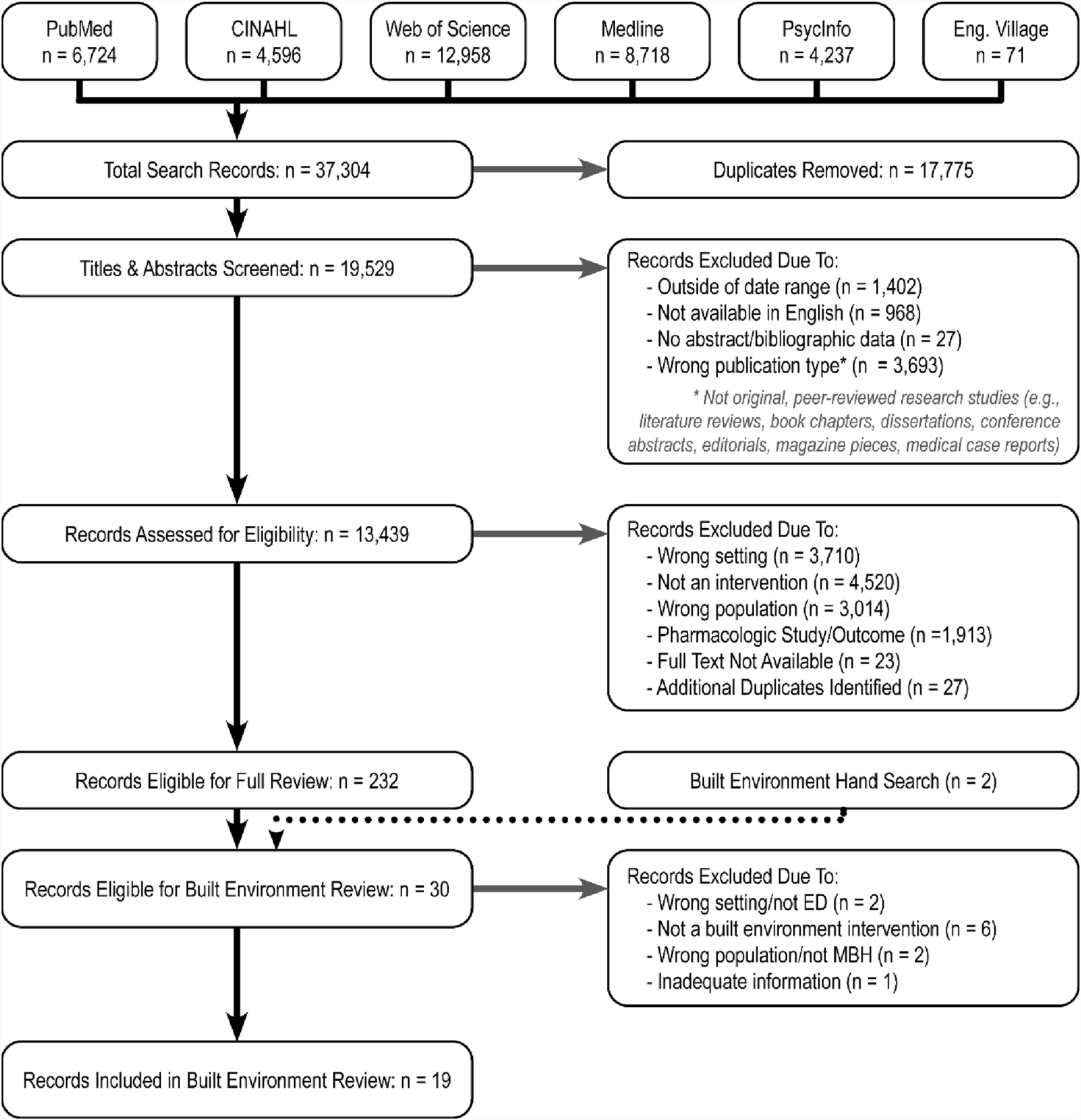
Review flowchart

The initial database search returned 37,304 entries. After compiling the results and removing duplicates (*n* = 17,775), two reviewers assessed the bibliographic data for the remaining 19,529 articles to remove any that did not meet preliminary eligibility criteria. These reviewers also removed articles whose titles or bibliographic data indicated they were literature reviews, editorials, or non-peer-reviewed or non-original research. Twelve reviewers read the titles and abstracts for the remaining 13,439 articles to determine their inclusion eligibility and assign labels for relevant SEIPS categories (Fig. 4). We identified 232 articles on ED interventions for the MBH caregiving process through the title and abstract review. Of these, 28 appeared to involve built environment interventions and moved forward to a full review. A hand search of reference lists in relevant literature reviews returned two additional papers not found in the database searches that met the inclusion criteria.

Next, two independent reviewers read each of the 30 built environment papers in full, assessed their inclusion eligibility, and evaluated their quality. Through this process, the reviewers jointly identified eleven articles that did not meet the inclusion criteria because of their focus on the wrong setting (e.g., inpatient unit) (Hasken et al., 2022; Johansson et al., 2018), wrong population (Cabilan et al., 2021; Cooke et al., 2007), not being a built environment intervention for MBH care (Daniel et al., 2021; Gillespie et al., 2013; Nicholas et al., 2020; Oblath et al., 2023; Strike et al., 2008; Woo et al., 2007), or inadequate information in the article to assess quality, methods, or outcomes (Grover & Lee, 2013).

**Fig. 5:**
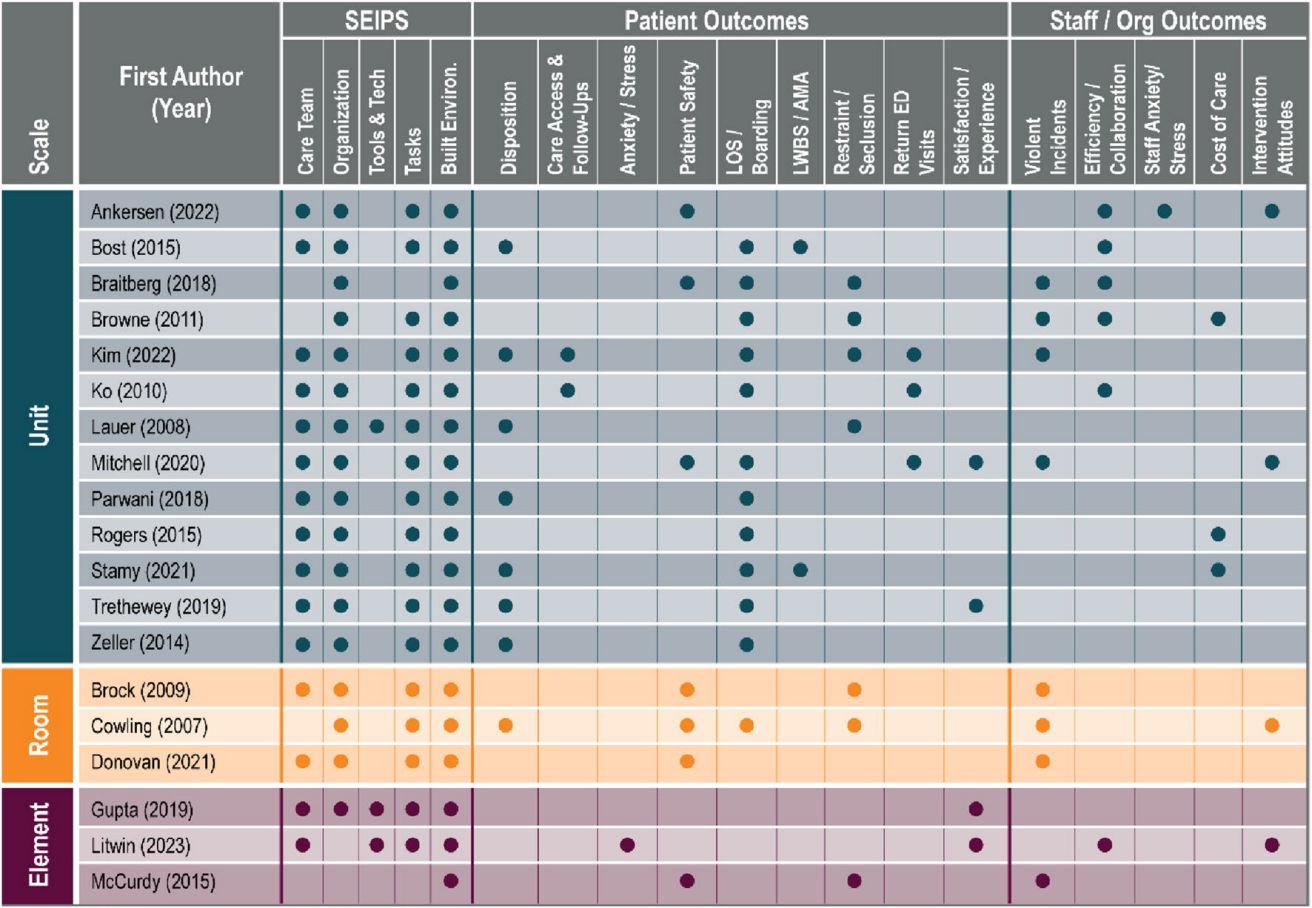
Examined SEIPS categories and outcomes by study

Table 2 provides details on the 19 included papers. Figure 5 summarizes the data. Of the 19 articles, most examined interventions in urban (*n* = 17), adult or general (*n* = 16) emergency departments. Most (*n* = 17) focused on interventions at single sites, while two looked at interventions across multiple EDs. Most interventions involved multiple SEIPS categories. Only one intervention exclusively involved the built environment (McCurdy et al., 2015). Nine studies came from the United States, five from Australia, two from Canada, and one from England, Denmark, and Hong Kong. The earliest publication was in 2007, with most published between 2018 and 2023.

**Table 2:** Data extraction from included studies

1st Author (year)	Title	Journal	Country	ED Setting	Intervention (Int.) Description	Int. Year	Study Objective(s)	Study Design/ Method(s)	
Ankersen (2022)	Bumpy Road: Implementing Integrated Psychiatric and Somatic Care in Joint-Specialty Emergency Departments: A Mixed-Method Study Using Normalization Process Theory	Journal of Integrated Care	Denmark	Single site Urban Adult/General	Addition of an integrated somatic/ psychiatric care pathway in an emergency department	2015	Understand staff barriers/ facilitators and patient outcomes of a psychiatric/ somatic integrated care pathway in an ED	Mixed methods Observation Staff focus groups Staff survey	
Bost (2015)	The Impact of a Flow Strategy for Patients Who Presented to an Australian Emergency Department with Mental Health Illness	International Emergency Nursing	Australia	Single site Urban Adult/General	Flow strategy change, including a short-stay ward for likely inpatient MBH admissions	2012	Evaluate the impact of a patient flow strategy on care delivery and outcomes	Quantitative Pre/post data analysis	
Braitberg (2018)	Behavioural Assessment Unit Improves Outcomes for Patients with Complex Psychosocial Needs	Emergency Medicine Australasia	Australia	Single site Urban Adult/General	Low-stimulus, 6-bed behavioral assessment unit located adjacent to an ED	2016	Assess a new care model, including an MBH assessment unit, on care outcomes	Quantitative Pre/post data analysis	
Brock (2009)	Use of a “Secure Room” and a Security Guard in the Management of the Violent, Aggressive, or Suicidal Patient in a Rural Hospital: A 3-Year Audit	Canadian Journal of Rural Medicine	Canada	Single site Rural Adult/General	Stripped-down, ground-floor “secure room” with a lockable door and observation windows monitored by a security guard	2004	Understand how physicians use the secure room and security guard system and patient dispositions	Quantitative Pre/post data analysis	
Browne (2011)	Improving the Care of Mentally Ill Patients in a Tertiary Emergency Department: Development of a Psychiatric Assessment & Planning Unit	Australasian Psychiatry	Australia	Single site Urban Adult/General	Psych. assessment and planning unit co-located in an acute inpatient MBH unit for patients waiting for inpatient admission	2007	Describe the impact of the new unit on MBH patient presentations, care, and outcomes	Quantitative Pre/post data analysis	
Cowling (2007)	Managing Acute Behavioural Disturbance in an Emergency Department Using a Behavioural Assessment Room	Australian Health Review	Australia	Single site Urban Adult/General	Containment area without any physical or removable hazards at the ED EMS entry for acutely behaviorally disturbed patients	2003	Audit the frequency and duration of the room’s use, patient characteristics, and staff experiences	Mixed Methods Retrospective data analysis Staff survey	
Donovan (2021)	Keeping Patients at Risk for Self-Harm Safe in the Emergency Department: A Protocolized Approach	The Joint Commission Journal on Quality and Patient Safety	United States	Single site Urban Adult/General	Safety improvements, including specially designed bathrooms, staff training, and high-risk patient protocols	2018	Assess the intervention’s impact on the number of attempted and actual self-harm episodes in the ED	Quantitative Pre/post data analysis	
Gupta (2019)	Utilization of a Novel Pathway in a Tertiary Pediatric Hospital to Meet the Sensory Needs of Acutely Ill Pediatric Patients	Frontiers in Pediatrics	United States	Single site Urban Pediatric	A “sensory pathway” that included staff training and sensory toolkits for use in the ED	2016	Evaluate the intervention’s impact on meeting patients’ sensory sensitivity needs	Quantitative Retrospective data analysis Surveys	
Kim (2022)	Emergency Psychiatric Assessment, Treatment, and Healing (EmPATH) Unit Decreases Hospital Admission for Patients Presenting with Suicidal Ideation in Rural America	Academic Emergency Medicine	United States	Single site Urban (serving rural area) Adult/General	Open concept EmPATH unit constructed adjacent to an ED with space for up to 12 patients	2018	Analyze the unit’s impact on hospital admissions for patients presenting with suicidal ideation or attempt	Quantitative Pre/post data analysis	
Ko (2010)	The Impact of Emergency Medicine Ward in Acute Intoxication Management	Hong Kong Journal of Emergency Medicine	Hong Kong	Single site Urban Adult/General	Emergency medicine ward for patients with acute intoxication	2007	Evaluate the ward’s impact on patient management, outcomes, and resource use	Quantitative Pre/post data analysis	
Lauer (2008)	Replacing the Revolving Door: A Collaborative Approach to Treating Individuals in Crisis	Journal of Psychosocial Nursing & Mental Health Services	United States	Single site Urban Adult/General	Secure unit within an ED designed to safely serve adults experiencing psychiatric crisis	2005	Evaluate the impact of the new unit on patient safety and disposition	Quantitative Pre/post data analysis	
Litwin (2023)	Designing a Sensory Kit to Improve the Environment for Children with Autism Spectrum Disorder in the Emergency Department	Journal of Autism and Developmental Disorders	Canada	Single site Urban Pediatric	Development of a low-cost sensory kit through an iterative participatory design process with children, parents, and healthcare providers	2018	Implement and refine a sensory toolkit to improve the ED experiences of children with Autism and their families	Mixed Methods Journey maps Observation Surveys	
McCurdy (2015)	Case Study: Design May Influence Use of Seclusion and Restraint	HERD: Health Environments Research & Design Journal	United States	Single site Urban Adult/General	Addition of a door between waiting and treatment areas in a psychiatric ED	2011	Examine the rates of seclusion and restraint before and after the door’s installation	Quantitative Pre/post data analysis	
Mitchell (2020)	The Efficacy, Safety & Acceptability of Emergency Embedded Psychiatric Assessment & Planning Units: An Evaluation of Psychiatry Assessment & Planning Units in Close Proximity to their Associated EDs	Australian & New Zealand Journal of Psychiatry	Australia	Multiple (3) sites Urban/suburban Adult/General	ED-embedded Psychiatric Assessment and Planning Units at three hospitals providing short-term care (< 72 h) for patients experiencing psychiatric crisis	2015	Evaluate the units in terms of patient and staff experiences, service safety and performance, and patient demographics	Mixed methods Interviews Pre/post data analysis	
Parwani (2018)	Opening of Psychiatric Observation Unit Eases Boarding Crisis	Academic Emergency Medicine	United States	Single site Urban Adult/General	Locked, 12-bed, psychiatric observation unit outside of the ED for adult patients	2013	Assess the unit’s effects on boarding, LOS, and inpatient psychiatric admission rates	Quantitative Pre/post data analysis	
Rogers (2015)	CARES: Improving the Care and Disposition of Psychiatric Patients in the Pediatric Emergency Department	Pediatric Emergency Care	United States	Single site Urban Pediatric	Emergency stabilization unit located on a separate campus for pediatric patient ED transfers	2007	Evaluate the effects of the rapid emergency stabilization unit on LOS and cost of care	Quantitative Pre/post data analysis	
Stamy (2021)	Economic Evaluation of the Emergency Department After Implementation of an Emergency Psychiatric Assessment, Treatment & Healing Unit	Academic Emergency Medicine	United States	Single site Urban Adult/General	Locked EmPATH unit located outside of an ED for up to 12 patients	2018	Determine the impact of the EmPATH unit on ED revenue, boarding time, and LOS	Quantitative Pre/post patient & financial data analysis	
Trethewey (2019)	Evaluation of the Psychiatric Decisions Unit (PDU): Effect on Emergency Department Presentations and Psychiatric Inpatient Admissions	Postgraduate Medical Journal	England	Single site Urban Adult/General	Non-bedded, acute mental health unit separate from the ED for up to 8 patients referred from the ED or Street Triage	2015	Evaluate the unit’s impact on ED presentations, inpatient psychiatric admissions, and patient satisfaction	Quantitative Pre/post data analysis Patient survey	
Zeller (2014)	Effects of a Dedicated Regional Psychiatric Emergency Service on Boarding of Psychiatric Patients in Area Emergency Departments	Western Journal of Emergency Medicine	United States	Multiple (5) sites Urban/suburban Adult/General	Crisis stabilization center serving 5 EDs in one county	2013	Examine boarding times and psychiatric hospitalization rates for five hospitals utilizing a crisis stabilization unit	Quantitative Retrospective data analysis	

### Critical Appraisal

**Fig. 6:**
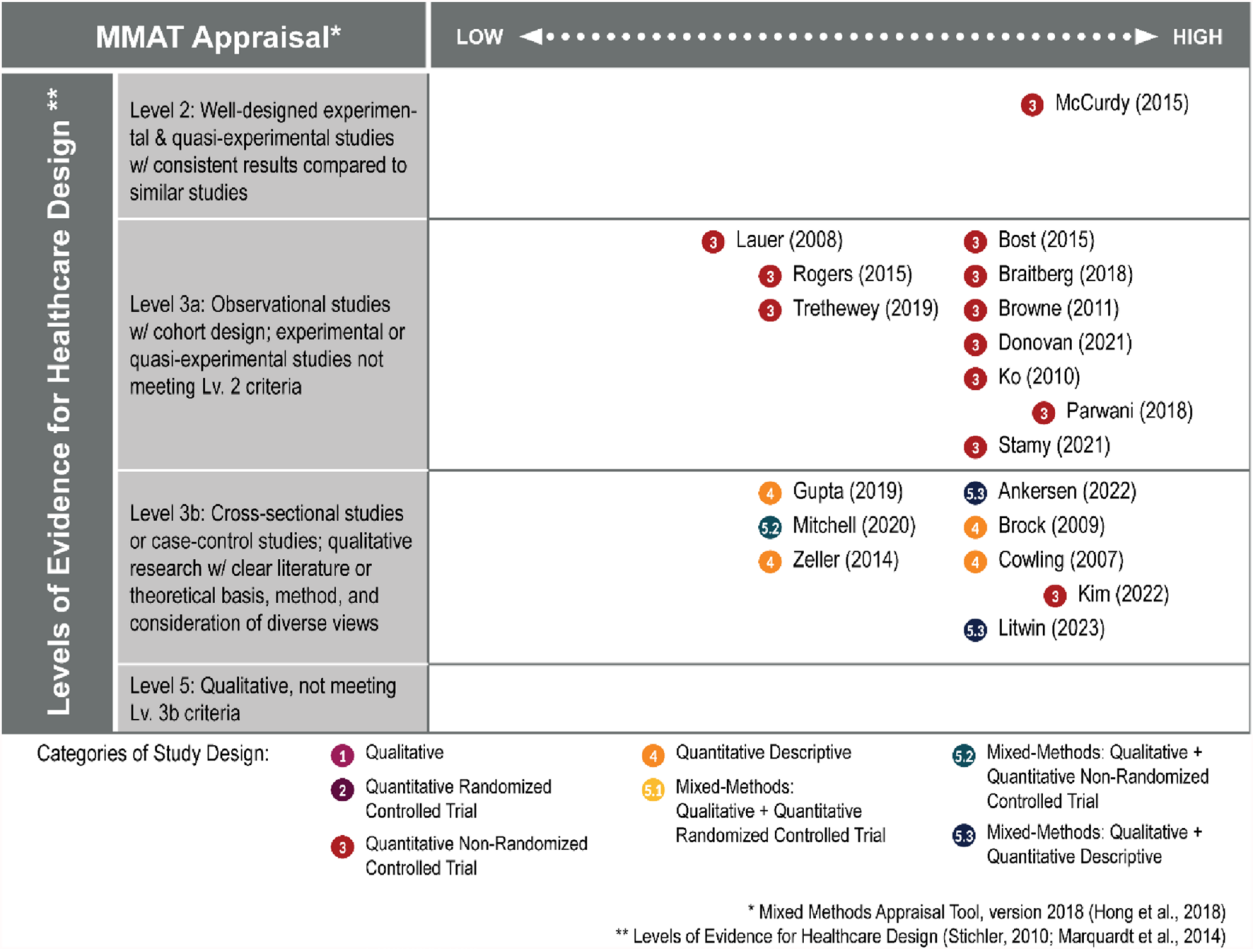
Relative MMAT scores and Levels of Evidence for the included studies

**Fig. 7:**
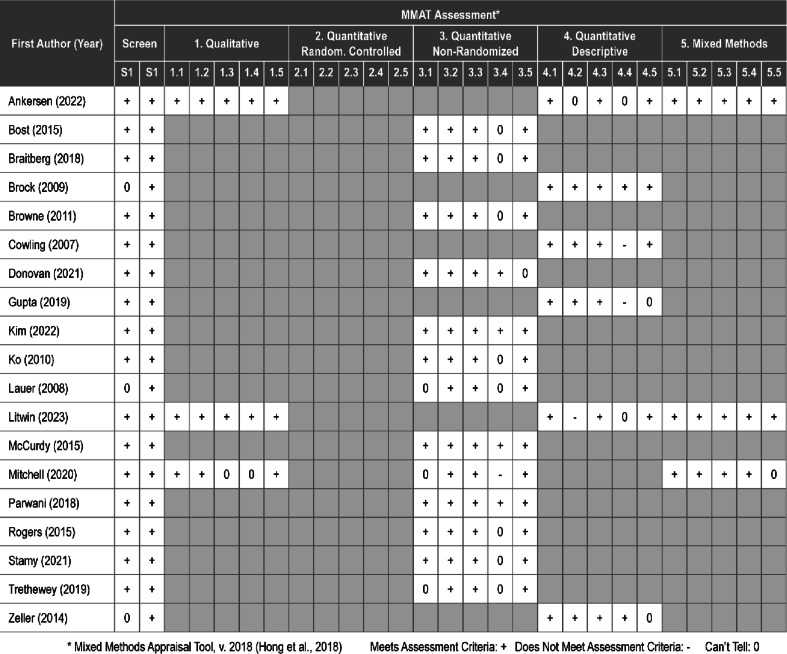
MMAT evaluation of the included studies

Per the Levels of Evidence for Healthcare Design model (Marquardt et al., 2014; Stichler, 2010), almost all the studies fell into categories 3a (*n* = 10) and 3b (*n* = 8), with one in category 2 (Fig. 6). Using the Mixed Methods Appraisal Tool (MMAT), version 2018 (Hong et al., 2018), most studies (*n* = 12) employed a quantitative, non-randomized study design (Fig. 7) and met most of the MMAT assessment criteria. Three of the included studies failed to specifically articulate their research questions (Brock et al., 2009; Lauer et al., 2008; Zeller et al., 2014), a screening requirement for the MMAT evaluation, but were retained for evaluation because both reviewers agreed the texts implicitly stated their objectives and aligned them with their methods. Furthermore, according to the recommendations outlined in the MMAT User Guide (Hong et al., 2018), the team did not exclude articles due to lower assessments on the MMAT. While the MMAT discourages calculating an overall score from the ratings of each criterion, several categories marked as unclear or not meeting the assessment criteria may indicate low methodological quality. All studies included in this review met MMAT criteria in more than half of the evaluation categories, suggesting they are of moderate to high methodological quality. As such, the review team is confident in the strength of the findings.

Three included studies employed mixed methods, none used qualitative methods alone, and none implemented quantitative randomized designs. Retrospective reviews of patient charts and hospital metrics were the most common data sources across all studies. Interventions fell into three scalar categories. Unit-scale interventions (*n* = 13) involved modifying or adding an entire unit. Room-scale interventions (*n* = 3) involved changing or adding a room or rooms associated with the ED. Element-scale interventions involved modifying, adding, or removing specific elements or individual features within a room or unit (*n* = 3). Outcomes focused on patients and staff, most commonly involving patient metrics like disposition, length of stay or boarding time, seclusion or restraint use, and safety incidents.

### Unit-Scale Interventions

Thirteen papers focused on unit-scale interventions, representing most of the included studies. Interventions typically involved creating new, dedicated units to divert or transfer 4–12 patients from the ED for short-stay assessment and holding (< 72 h). Some EDs added units adjacent to or within emergency departments (Bost et al., 2015; Braitberg et al., 2018; Kim et al., 2022; Ko et al., 2010; Lauer & Brownstein, 2008; Mitchell et al., 2020). Others incorporated them within the same hospital or campus (Browne et al., 2011; Parwani et al., 2018; Stamy et al., 2021). Still others created off-campus locations serving one or more hospitals (Rogers et al., 2015; Trethewey et al., 2019; Zeller et al., 2014). Rather than adding a new unit, one ED created an integrated care model for treating MBH and non-MBH patients in the same ED to help increase psychiatric care access (Ankersen et al., 2022). In addition to the built environment, interventions at the unit scale typically involved adding dedicated psychiatric staff or training ED staff to work with MBH patients and patient flow/process changes to triage, transfer, assess, and observe patients experiencing MBH crises.

All but one of the unit-scale studies described post-intervention quantitative data from hospital metrics and electronic medical records (EMRs) or compared EMR data before and after the intervention. Unlike the other unit-scale studies, Ankersen et al. (2022) used observations, interviews, focus groups, and a questionnaire to explore ED staff perceptions of the intervention after its implementation. Mitchell et al. (2020) employed staff and patient interviews, surveys, and EMR data to assess an intervention’s impact. Length of stay and patient disposition were among the most frequently examined outcomes. Other outcomes included safety and security, efficiency and cost, and satisfaction and experience.

#### Length of Stay

Eleven of the thirteen unit-scale studies examined intervention impacts on patient length of stay (LOS) and boarding (Bost et al., 2015; Braitberg et al., 2018; Browne et al., 2011; Kim et al., 2022; Ko et al., 2010; Mitchell et al., 2020; Parwani et al., 2018; Rogers et al., 2015; Stamy et al., 2021; Trethewey et al., 2019; Zeller et al., 2014). Most found that adding an emergency MBH unit decreased LOS and boarding times.

For example, Braitberg et al. (2018) found that average LOS decreased from 328 to 180 min after opening a 6-bed behavioral assessment unit co-located with an urban adult ED in Australia. A study of a 12-bed adjacent psychiatric observation unit that allowed moving MBH patients out of the main ED while awaiting assessment or disposition found significant LOS decreases for non-MBH patients (Parwani et al., 2018). Studies on transferring MBH patients from EDs to separate locations for emergency psychiatric assessment, disposition, and boarding also found significant decreases in ED boarding times associated with the interventions (Rogers et al., 2015; Trethewey et al., 2019; Zeller et al., 2014). Likewise, Stamy et al. (2021) found that transferring non-violent patients in psychiatric crisis from an ED to an EmPATH (Emergency Psychiatric Assessment, Treatment, and Healing) unit – a separate acute care unit designed to stabilize patients in a therapeutic milieu – in the same hospital decreased psychiatric patients’ median LOS from 351 to 334 min and boarding times from 212 to 152 min. However, they did not find decreases in boarding time or LOS for non-psychiatric patients.

Still, not all studies have found LOS decreases associated with adding units for MBH care. For example, a study of three ED-adjacent Psychiatric Assessment and Planning Units (PAPUs) in Australia found a decrease in LOS at only one ED and statistically significant increases at the other two (Mitchell et al., 2020). A patient flow strategy involving a short-stay ward for patients in psychiatric crisis found only non-significant decreases in average LOS (Bost et al., 2015).

#### Disposition, Returns, and Follow-Up

Seven studies examined unit-scale interventions’ impacts on patient dispositions – plans for or destination of a patient after leaving the ED – with most interventions relating to decreases in inpatient psychiatric admissions. For example, a pre/post-intervention study by Kim et al. (2022) found that a 12-patient EmPATH unit located away from the ED but in the same hospital decreased inpatient psychiatric admissions (pre: 57.1% vs. post: 27.3%) and inpatient admissions of any type (pre: 65% vs. post: 42.3%) for patients with suicidal ideation or attempt.

Opening a CAPES (Crisis Assessment and Psychiatric Services) Unit – a secure area within an ED serving adult patients in psychiatric crisis – related to a 43% decline in involuntary psychiatric inpatient admissions at an urban ED in the United States (Lauer & Brownstein, 2008). Similarly, a 12-bed psychiatric unit outside another ED helped decrease inpatient psychiatric admission rates from 42% to 25.4% (Parwani et al., 2018). Units located off-site or off-campus from the ED, including an 8-patient Psychiatric Decision Unit serving a community in the United Kingdom (Trethewey et al., 2019) and a Psychiatric Emergency unit serving a county in California (Zeller et al., 2014), also contributed to decreases in inpatient psychiatric admissions, with the California unit reducing inpatient hospitalization by as much as 75% for the county compared to other counties in the state. Still, not all studies found similar changes in patient disposition related to unit-scale interventions. Bost et al. (2015) found that disposition locations and rates did not change with a new short-stay unit for MBH patients.

Studies by Kim et al. (2022) and Ko et al. (2010) investigated the impact of interventions involving psychiatric emergency units added adjacent to medical EDs – including specialized psychiatric staff, training, and organizational policies – on patient follow-up after an ED presentation and found that the interventions were associated with a 60% increase in MBH follow-up appointments scheduled within 30 days of discharge (Kim et al., 2022) and a significantly higher proportion of acutely intoxicated patients receiving psychiatric care (Ko et al., 2010). Ko et al. (2010) did not find a significant difference in 28-day readmissions following the intervention, but Kim et al. (2022) and Mitchell et al. (2020) found that readmission decreased post-intervention. Unit-scale interventions also reduced instances of leaving without being seen (LWBS) and leaving against medical advice (AMA) in two studies (Bost et al., 2015; Stamy et al., 2021).

#### Safety and Security

Five studies investigated intervention impacts on patient and staff safety and security, including instances of restraint or seclusion and security code events related to aggressive, violent, or behaviorally disturbed patients. Kim et al. (2022) found no significant difference in the proportion of restraint use post-intervention. Still, Braitberg et al. (2018), Lauer and Brownstein (2008), and Browne et al. (2011) found significant decreases in restraint use and average restraint time associated with their interventions. Moreover, while Kim et al. (2022) also found no significant difference in the proportion of security code initiations before and after the opening of an ED-adjacent EmPATH unit, Braitberg et al. (2018) and Browne et al. (2011) identified significant decreases in the periods after their respective interventions. The discrepancy in findings by Kim et al. (2022) compared to the other studies could be due to their exclusive focus on patients with suicidal ideation and attempt.

#### Efficiency and Cost

Three studies examined costs associated with unit-scale interventions. Stamy et al. (2021) found that a locked, 12-patient EmPATH unit located away from the ED but in the same hospital increased efficiencies, contributing to over $800,000 in additional annual revenues post-intervention. However, cost analysis indicated that the increased revenue only covered half the cost of planning, constructing, and operating the unit. Rogers et al. (2015) found significant decreases in ED charges and payments per patient after opening a pediatric emergency stabilization unit. Still, the researchers found that, although gross revenues declined, efficiency improvements associated with the intervention netted dramatic overall cost savings per patient. Likewise, Browne et al. (2011) found that the safer physical environment in a unit for patients boarding for more than 24 h or awaiting inpatient admission reduced one-to-one patient monitoring needs, contributing to cost savings over the study period.

#### Satisfaction and Experience

Only two studies assessed ED staff’s perceptions of unit-scale interventions. Mitchell et al. (2020) found that staff at three studied EDs had favorable perceptions of MBH assessment units added adjacent to their EDs. Another study of an integrated care intervention that blended medical and psychiatric care found that staff disagreed with the intervention (64%) because they did not see the ED as an appropriate, safe, or therapeutic place for psychiatric patients due to unsafe equipment and objects, and its noisy, stressful atmosphere.

### Room-Scale Interventions

Three studies examined room-scale interventions (Brock et al., 2009; Cowling et al., 2007; Donovan et al., 2021), all to improve patient and staff safety. Brock et al. (2009) and Cowling et al. (2007) examined new, secure rooms within emergency departments. Meanwhile, Donovan et al. (2021) studied a quality improvement project that included safety-focused renovations of ED bathrooms.

The “secure room” described by Brock et al. (2009) was a lockable, ground-floor room with only a bed, observation windows, and a security guard in a rural Canadian hospital that aimed to improve patient safety and reduce staffing and costs associated with the hospital’s former strategy of holding patients in ED rooms before transferring them over 200 km to the nearest psychiatric referral center. A 3-year retrospective chart audit indicated that the hospital used the room about once per month, primarily for patients experiencing depression, drug overdose, or self-harm risk. 80% of patients admitted to the secure room were stabilized and discharged locally within two nights without referral or transfer. Although the study did not compare collected data to pre-intervention data, the team assessed the room as a successful and feasible intervention for a small, rural hospital to efficiently and cost-effectively manage MBH patients.

Similarly, Cowling et al. (2007) retrospectively analyzed EMR data to assess outcomes associated with constructing and using a Behavioral Assessment Room (BAR) at an urban hospital in Australia. The BAR, located at the periphery of the ED with direct access to the EMS/police entry, lacks any removable or physical hazards and has an extra-wide doorway and observation windows. It facilitates removing acutely behaviorally disturbed patients from the public ED environment for safety. A 1-year retrospective chart audit indicated the room was used for 0.4% of all patients during the study period and, similar to the room studied by Brock et al. (2009), was mainly used for substance-related disturbances. The average patient stay in the BAR was only 20 min. ED staff surveys indicated that 98.5% thought it made the ED safer, and 74.5% thought it improved the ED’s management of acutely disturbed patients.

Donovan et al. (2021) analyzed the impacts of an organizational safety improvement initiative at an urban academic medical center that included policy and training interventions and built environment improvements in patient bathrooms. The updated bathrooms included shatterproof fixtures and mirrors, paper waste bin liners, minimal ligature risks, and mirrors and curtains that supported patient monitoring while balancing privacy. Compared to pre-intervention data, patients experienced significantly longer lengths of stay after the intervention but fewer incidents of attempted self-harm (pre: 2.95/ 1,000 patients; post: 1.33/1,000 patients) and non-life-threatening self-harm (pre: 1.36/1000 patients; post: 0.22/1000 patients). Such decreases are clinically (though not statistically) significant and illustrate that policy and training interventions combined with built environment improvements can increase patient safety in EDs.

### Element-Scale Interventions

Three studies examined element-scale interventions (Gupta et al., 2019; Litwin & Sellen, 2023; McCurdy et al., 2015). A study by McCurdy et al. (2015) was the only one that isolated and examined a built environment intervention. In this study, the researchers investigated the impact of adding a door between the waiting and treatment areas of a dedicated emergency psychiatric unit adjacent to an ED. Although the unit also instituted the use of the Brøset scale – a scale for predicting violence – the team collected data before and after the scale’s institution and before and after the installation of the door to isolate the door’s impact on assaults and seclusion/restraint incidents, controlling for the separate task/organization intervention. Adding the door did not change the number of assaults – there were none before or after the intervention – but did significantly lower the number of incidents of seclusion and restraint (pre: 0.032/1000; post: 0.018/1000). The research team attributed this reduction to limiting patient movement throughout the unit and associated improvements in security’s ability to visualize patients and quickly deter potential incidents.

The other two element-scale interventions involved sensory toolkits to moderate the ED environment, improve patient satisfaction and experiences, and reduce stress and anxiety (Gupta et al., 2019; Litwin & Sellen, 2023). Both sensory kits included items like sound machines, lights, and bubble towers to help moderate the (usually highly stimulating) ED sensory environment. Staff feedback obtained by Litwin and Sellen (2023) indicated that successful sensory kits should “transform the room.” Both interventions included staff training, revised protocols for patients with sensory sensitivities, and signage to notify families and patients of the toolkits’ availability. Family surveys in both studies indicated that families were satisfied with the sensory kits and thought they improved patients’ care experience. Staff surveys by Litwin and Sellen (2023) suggested that the time required for staff to retrieve the kit was one of the most significant barriers to implementation.

## Discussion

The themes and findings that emerged from the 19 included studies in this review suggest that, although challenges faced in emergency MBH care are complex and involve numerous work systems, changes to the built environment at the unit, room, or element scale can contribute to systems-based ED interventions and improve care processes, safety, and experiences for patients and staff. Studies show that many healthcare providers and organizations are enthusiastic and ready to implement changes to address issues related to emergency MBH care, but face significant resource constraints and other barriers to implementing interventions (Bowden et al., 2022). As evidenced by the intervention studies included in this review, the challenges targeted by such interventions are complex and necessitate changes beyond just the built environment to make a meaningful impact. Still, this review’s findings highlight numerous implications and opportunities for research, design, and healthcare practice.

### Research Implications

Most reviewed studies examined EMR and retrospective hospital data in their inquiry. Few studies collected data directly from patients, families, staff, or observations. While retrospective data analysis helps quantify impacts that healthcare organizations can use to make informed business decisions, it cannot provide much insight into the lived experiences or perspectives of stakeholders. In the broader literature, numerous studies have explored the experiences of patients, families, and staff in relation to the built environment (e.g., studies outlined in literature reviews by Bull et al. (2024) and Gripko et al. (2023). However, within the narrow focus of this review – built environment interventions in emergency departments – such perspectives were not well-represented. Accordingly, future studies of ED interventions could use other data sources and methods to capture data directly from those experiencing the intervention. Such an approach could provide a more balanced perspective and situate an intervention within its clinical and social context.

Seventeen of the studies focused exclusively on interventions at single EDs. Although such studies provide valuable data and insights, single cases limit generalizability (Yin, 2018). This review identified variation in outcomes between similar interventions, such as in impacts to LOS of MBH units added within or adjacent to existing EDs (Bost et al., 2015; Braitberg et al., 2018; Kim et al., 2022; Ko et al., 2010; Lauer & Brownstein, 2008). However, because each of these studies implemented their built environmental changes in conjunction with new processes, training, and other interventions, and explored varied data and different outcomes, it is not possible to compare them directly across the studies. Future research could assess interventions across multiple EDs or replicate previous intervention studies to compare implementations and outcomes.

Likewise, only three studies examined interventions in pediatric EDs, and two looked at non-urban (one suburban, one rural) EDs. Still, rural ED visit rates are increasing faster than urban ED visit rates (38), and MBH needs in children and adolescents are rising quickly (Bommersbach et al., 2023). Accordingly, more research is needed in non-adult and non-urban environments. Lastly, only one study isolated a specific design element (McCurdy et al., 2015) and analyzed associated outcomes. Although emergency MBH care is complex and involves multiple work systems, future research that isolates specific built environmental features and tests their impacts on critical metrics (like safety or LOS) could help develop an evidence base for future design decisions.

### Design Implications

These studies illustrate that unit-scale interventions – including adding dedicated units adjacent to or embedded within an ED, in a separate area within the hospital, or away from the hospital – may help alleviate ED crowding and backup by allowing MBH patients to quickly transfer out of the ED instead of spending long periods waiting (Table 3). Alleviating crowding and backups is critical because long waiting and boarding times contribute to poor outcomes, care delays, workflow inefficiencies, and capacity concerns arising from a misalignment between patient needs and environment and resource allocation (Claudius et al., 2014; Feuer et al., 2023; Leyenaar et al., 2021) and relate to diminished perceptions of care and increased safety concerns (Foster et al., 2021; McCarty et al., 2022). Most included studies (though not all) found that unit-scale interventions related to decreases in LOS and boarding times for MBH and non-MBH patients. Several studies also found that such unit-scale interventions decreased the proportion of patients admitted to inpatient psychiatric care, instances of patients leaving without being seen or against medical advice, and readmissions within the month following the ED visit.

Dedicated MBH units, as described in the studies, were typically designed to be safer and calmer environments for patients in crisis. Many ED environments lack safe accommodations to help patients cope, leaving them focused on distressing thoughts and worries and contributing to escalation that can result in physical or chemical restraint (Dalton et al., 2023). However, the studies included in this review suggest that calmer, safer, and more therapeutic dedicated MBH units are associated with fewer instances of restraint use, security codes, and other safety incidents. Although constructing these units can be expensive, the associated efficiency and safety improvements may make dedicated units an attractive option for healthcare organizations seeking to improve efficiency, patient and staff safety, and patient satisfaction.

At the room scale, interventions that minimize physical safety risks for patients in acute MBH crises can improve patient and staff safety and support de-escalation and stabilization efforts. Although room-scale interventions may be implemented in all types of EDs, these smaller-scale interventions can be practical strategies for small or rural hospitals that may not have the space or resources for dedicated units, as illustrated by Brock et al. (2009).

Few studies examined element-scale interventions embedded or designed into the built environment. Gupta et al. (2019) and Litwin and Sellen (2023) assessed the implementation of sensory kits to moderate the ED environment for patients with sensory sensitivities. Both found that the sensory kits improved patients’ and families’ ED experiences. Still, they only used mobile elements, not elements integrated into the physical environment. Opportunities exist for designers to incorporate sensory control and customization into ED treatment and waiting areas – like controllable lighting or sound systems – and test their implementation and outcomes.

### Clinical Practice Implications

This systematic review is one part of a larger review examining ED-based interventions for improving mental and behavioral health care. Of the 232 studies identified in that larger endeavor, only 19 involved built environmental interventions. Many studies, including those with built environment components, incorporated changes to patient flows, clinical processes, staffing, and training that contributed to the observed outcomes in the studies. Since this review focused on interventions to the physical environment for emergency MBH care, we did not evaluate the impacts of clinical and operational interventions. Our team is working on additional sub-reviews that will examine literature related to other ED work systems.

Although healthcare is complex and involves multiple work systems, research shows that built environments can and do affect healthcare delivery, safety, and experiences (e.g., Bull et al., 2024; Gripko et al., 2023; Rowe & Knox, 2023; Ulrich et al., 2008). While ED-based MBH interventions rarely isolate the built environment, the nineteen studies in this review illustrate how physical environmental changes can support, complement, or contribute to systems-based approaches for improving patient and staff safety and experiences in emergency MBH care.

**Table 3:** Impacts and applications of included studies

Scale	Strategy (*n* studies)	Referenced Studies		Impact	Tested Setting(s)	Possible Setting(s)
Unit	Unit adjacent to or within the ED *(6)*	Bost (2015) Braitberg (2018) Kim (2022) Ko (2010) Lauer (2008) Mitchell (2020)	* Impact Varied * • Four (4) studies found decreased LOS and boarding times • Two (2) studies found insignificant decreases in LOS or significant increases in LOS post-intervention • Two (2) studies found reductions in inpatient admissions and reduced readmission rates • Three (3) studies noted decreased restraint use/duration		Urban Suburban	Urban Suburban Rural
	Unit within the same hospital or campus *(3)*	Browne (2011) Parwani (2018) Stamy (2021)	* Impact Varied * • Dedicated MBH units in the hospital or campus related to significant ED boarding time reductions • One (1) study found a reduction in inpatient admissions • One (1) study noted decreased restraint use/duration		Urban	Urban Suburban
	Off-campus location serving 1 + hospitals *(3)*	Rogers (2015) Trethewey (2019) Zeller (2014)	MBH units located off-site, or off-campus, contributed to decreases in inpatient psychiatric admissions		Urban	Urban Suburban Rural
	Integrated care model: MBH and non-MBH patients in the same ED *(1)*	Ankersen (2022)	Integration/adoption of a unit-wide model was challenging • Psychiatric nurse in the ED facilitated integrated care • Acute psychiatric treatment area was noisy, making it difficult for nurses to concentrate and interrupting conversations with patients		Urban	Urban Suburban
Room	Secure room *(1)*	Brock (2009)	The secure room decreased MBH transfers and admissions to other facilities by 80%		Rural	Urban Suburban Rural
	Behavioral assessment room *(1)*	Cowling (2007)	Staff thought the room made the ED safer and improved patient management • Average room LOS was 20 min		Urban	Urban Suburban Rural
	Safer bathrooms *(1)*	Donovan (2021)	LOS significantly increased after the broader intervention (including bathroom improvements), but incidents of attempted self-harm decreased		Urban	Urban Suburban Rural
Element	Sensory kits *(2)*	Gupta (2019) Litwin (2023)	Families were satisfied with the sensory kits and thought they improved patients’ experiences		Urban	Urban Suburban Rural
	Secure door *(1)*	McCurdy (2015)	Adding the door to separate waiting and treatment significantly lowered the number of incidents of seclusion and restraint		Urban	Urban Suburban Rural

### Limitations

Though the review team made every effort to capture the body of relevant literature and assess the findings unbiasedly, this review has limitations. We carefully constructed keywords and searched six databases, but we may have missed some literature due to the nature of our keywords or the chosen databases. We constructed our review process and trained the review team to assess records consistently and with minimal bias, but some relevant papers may have been excluded due to the limitation of making an initial decision solely from the title and abstract, which may not have fully or accurately captured the nature and content of the study.

Furthermore, this review limited inclusion eligibility to reports published in English and available in full via open access or our library subscriptions. This criterion may have excluded some relevant studies or biased the findings toward English-speaking (mostly North American) populations and contexts. We also limited our search to include only research published since 2000, which may exclude some relevant studies. Moreover, despite the review team’s efforts to mitigate bias by employing widely accepted assessment criteria and multiple reviewers, it is impossible to eliminate all biases.

## Conclusions

The ED built environment provides the context and setting for care delivery, influences workflows, and shapes user interactions, but it can be counter-therapeutic for patients in MBH crisis. With ED MBH presentation rates rising, findings from 19 studies suggest that changes to the built environment at the unit, room, or element scale can contribute to systems-based interventions addressing challenges related to emergency MBH caregiving, thereby improving care processes, safety, and experiences for patients and staff. However, more research is needed to capture patient and staff voices, examine various types of EDs, and compare interventions across sites. Through future research and design innovation, healthcare organizations and designers can create built environments that enhance safety, facilitate high-quality care, and support the health and well-being of all involved in emergency MBH care.

## Supplementary Information

Below is the link to the electronic supplementary material.

Supplementary Material 1

## Data Availability

No datasets were generated or analysed during the current study.
