# Supplementary material for: A Systematic Review of Built Environment Interventions to Improve Patient and Staff Outcomes in Emergency Department Mental and Behavioral Healthcare
Source: Adm Policy Ment Health. 2026 Apr 7;53(4):370–87. doi: 10.1007/s10488-026-01502-0 (PMC13191604; doi:10.1007/s10488-026-01502-0)
Supplement: Supplementary file 1 — Supplementary Material 1 [file 10488_2026_1502_MOESM1_ESM.pdf]

PubMed		
A) Tasks & Organizational Conditions	Setting	((emergency service, hospital[MeSH Terms]) OR (emergency services, psychiatric[MeSH Terms]) OR ("emergency department"[Title/Abstract]) OR ("emergency room"[Title/Abstract]) OR ("emergency service"[Title/Abstract]) OR ("emergency ward"[Title/Abstract]) OR ("emergency clinic"[Title/Abstract]) OR ("emergency unit"[Title/Abstract]) OR ("accident and emergency"[Title/Abstract]))
	Population	**AND** ((("mental health"[MeSH Terms]) OR (behavioral medicine[MeSH Terms]) OR (mental disorders[MeSH Terms]) OR (behavioral medicine[MeSH Terms]) OR ("mental health"[Title/Abstract]) OR ("behavioral health"[Title/Abstract]) OR ("psychiatry"[Title/Abstract]) OR ("psychology"[Title/Abstract]) OR ("psychiatric"[Title/Abstract]) OR ("autism"[Title/Abstract]) OR ("autism spectrum"[Title/Abstract]) OR ("cognitive impairment"[Title/Abstract]) OR ("intellectual impairment"[Title/Abstract]) OR ("cognitively impaired"[Title/Abstract]) OR ("intellectually impaired"[Title/Abstract]) OR ("depression"[Title/Abstract]) OR ("anxiety"[Title/Abstract]) OR ("attention deficit"[Title/Abstract]) OR ("hyperactivity"[Title/Abstract]) OR ("adhd"[Title/Abstract]) OR ("post traumatic stress"[Title/Abstract]) OR ("ptsd"[Title/Abstract]) OR ("obsessive compulsive"[Title/Abstract]) OR ("tourette"[Title/Abstract]) OR ("oppositional defiant"[Title/Abstract]) OR ("mania"[Title/Abstract]) OR ("manic"[Title/Abstract]) OR ("depressive"[Title/Abstract]) OR ("suicidal"[Title/Abstract]) OR ("schizophrenia"[Title/Abstract]) OR ("schizophrenic"[Title/Abstract]) OR (“eating disorder”[Title/Abstract]) OR (“anorexia”[Title/Abstract]) OR (“bulimia”[Title/Abstract]) OR (substance-related disorders[MeSH Terms]) OR (“addiction”[Title/Abstract]) OR (“addicted”[Title/Abstract]) OR (“overdose”[Title/Abstract]) OR (“substance abuse”[Title/Abstract]) OR (“alcohol abuse”[Title/Abstract]) OR (“drug abuse”[Title/Abstract]) OR (“intoxication”[Title/Abstract]) OR (“intoxicated”[Title/Abstract])) **AND** ((emergency nursing[MeSH Terms]) OR (emergency medicine[MeSH Terms]) OR (psychology[MeSH Terms]) OR (psychiatry[MeSH Terms]) OR (patients[MeSH Terms]) OR (family[MeSH Terms]) OR (parents[MeSH Terms]) OR (caregivers[MeSH Terms]) OR ("patient"[Title/Abstract]) OR ("doctor"[Title/Abstract]) OR ("physician"[Title/Abstract]) OR ("nurse"[Title/Abstract]) OR ("clinician"[Title/Abstract]) OR ("healthcare worker"[Title/Abstract]) OR ("psychiatrist"[Title/Abstract]) OR ("psychologist"[Title/Abstract]) OR ("social worker"[Title/Abstract]) OR ("counselor"[Title/Abstract]) OR ("therapist"[Title/Abstract]) OR ("care team"[Title/Abstract]) OR ("security"[Title/Abstract]) OR ("ems"[Title/Abstract]) OR (“family”[Title/Abstract]) OR (“parents”[Title/Abstract]) OR (“caregivers”[Title/Abstract])))
	Intervention	**AND** ((education, continuing[MeSH Terms]) OR (education, medical[MeSH Terms]) OR (education, nursing[MeSH Terms]) OR (workflow[MeSH Terms]) OR (decision making[MeSH Terms]) OR ("task"[Title/Abstract]) OR ("process"[Title/Abstract]) OR ("workflow"[Title/Abstract]) OR ("triage"[Title/Abstract]) OR ("fast track"[Title/Abstract]) OR ("lean"[Title/Abstract]) OR ("treatment"[Title/Abstract]) OR ("assess"[Title/Abstract]) OR ("assessment"[Title/Abstract]) OR ("screening"[Title/Abstract]) OR ("medical screening"[Title/Abstract]) OR ("psychiatric screening"[Title/Abstract]) OR ("evaluation"[Title/Abstract]) OR ("evaluate"[Title/Abstract]) OR ("training"[Title/Abstract]) OR ("professional development"[Title/Abstract]) OR ("mentor"[Title/Abstract]) OR ("mentorship"[Title/Abstract]) OR ("learning"[Title/Abstract]) OR ("education"[Title/Abstract]) OR ("culture"[Title/Abstract]) OR ("management"[Title/Abstract]) OR ("manager"[Title/Abstract]) OR ("change"[Title/Abstract]) OR ("leader"[Title/Abstract]) OR

		("leadership"[Title/Abstract]) OR ("team"[Title/Abstract]) OR ("teamwork"[Title/Abstract]) OR ("policy"[Title/Abstract]) OR ("policies"[Title/Abstract]) OR ("staffing"[Title/Abstract]) OR ("scheduling"[Title/Abstract]) OR ("schedule"[Title/Abstract]))
	Outcome	**AND** ((defense mechanisms[MeSH Terms]) OR (emotions[MeSH Terms]) OR (behavioral symptoms[MeSH Terms]) OR (length of stay[MeSH Terms]) OR (patient participation[MeSH Terms]) OR (family relationships, professional[MeSH Terms]) OR ("aggression"[Title/Abstract]) OR ("aggressive"[Title/Abstract]) OR ("agitation"[Title/Abstract]) OR ("agitated"[Title/Abstract]) OR ("confusion"[Title/Abstract]) OR ("distress"[Title/Abstract]) OR ("anger"[Title/Abstract]) OR ("angry"[Title/Abstract]) OR ("comfort"[Title/Abstract]) OR ("discomfort"[Title/Abstract]) OR ("pain"[Title/Abstract]) OR ("satisfaction"[Title/Abstract]) OR ("dissatisfaction"[Title/Abstract]) OR ("privacy"[Title/Abstract]) OR ("private"[Title/Abstract]) OR ("safe"[Title/Abstract]) OR ("safety"[Title/Abstract]) OR ("secure"[Title/Abstract]) OR ("security"[Title/Abstract]) OR ("violent"[Title/Abstract]) OR ("violence"[Title/Abstract]) OR ("anxiety"[Title/Abstract]) OR ("anxious"[Title/Abstract]) OR ("stress"[Title/Abstract]) OR ("strain"[Title/Abstract]) OR ("self harm"[Title/Abstract]) OR ("self injury"[Title/Abstract]) OR ("suicide"[Title/Abstract]) OR ("suicidal"[Title/Abstract]) OR ("sedation"[Title/Abstract]) OR ("sedate"[Title/Abstract]) OR ("sedative"[Title/Abstract]) OR ("restrain"[Title/Abstract]) OR ("restraint"[Title/Abstract]) OR ("ligature"[Title/Abstract]) OR ("risk"[Title/Abstract]) OR ("resilience"[Title/Abstract]) OR ("length of stay"[Title/Abstract]) OR ("leaving without being seen"[Title/Abstract]) OR ("lwbs"[Title/Abstract]) OR ("elope"[Title/Abstract]) OR ("elopement"[Title/Abstract]) OR ("injure"[Title/Abstract]) OR ("injury"[Title/Abstract]) OR ("family engagement"[Title/Abstract]) OR ("transfer"[Title/Abstract]) OR (“wellbeing"[Title/Abstract]) OR (“outpatient follow up"[Title/Abstract]) OR (“ambulatory follow up"[Title/Abstract]))
B) Built Environment	Setting	((emergency service, hospital[MeSH Terms]) OR (emergency services, psychiatric[MeSH Terms]) OR ("emergency department"[Title/Abstract]) OR ("emergency room"[Title/Abstract]) OR ("emergency service"[Title/Abstract]) OR ("emergency ward"[Title/Abstract]) OR ("emergency clinic"[Title/Abstract]) OR ("emergency unit"[Title/Abstract]) OR ("accident and emergency"[Title/Abstract]))
	Population	**AND** ((("mental health"[MeSH Terms]) OR (behavioral medicine[MeSH Terms]) OR (mental disorders[MeSH Terms]) OR (behavioral medicine[MeSH Terms]) OR ("mental health"[Title/Abstract]) OR ("behavioral health"[Title/Abstract]) OR ("psychiatry"[Title/Abstract]) OR ("psychology"[Title/Abstract]) OR ("psychiatric"[Title/Abstract]) OR ("autism"[Title/Abstract]) OR ("autism spectrum"[Title/Abstract]) OR ("cognitive impairment"[Title/Abstract]) OR ("intellectual impairment"[Title/Abstract]) OR ("cognitively impaired"[Title/Abstract]) OR ("intellectually impaired"[Title/Abstract]) OR ("depression"[Title/Abstract]) OR ("anxiety"[Title/Abstract]) OR ("attention deficit"[Title/Abstract]) OR ("hyperactivity"[Title/Abstract]) OR ("adhd"[Title/Abstract]) OR ("post traumatic stress"[Title/Abstract]) OR ("ptsd"[Title/Abstract]) OR ("obsessive compulsive"[Title/Abstract]) OR ("tourette"[Title/Abstract]) OR ("oppositional defiant"[Title/Abstract]) OR ("mania"[Title/Abstract]) OR ("manic"[Title/Abstract]) OR ("depressive"[Title/Abstract]) OR ("suicidal"[Title/Abstract]) OR ("schizophrenia"[Title/Abstract]) OR ("schizophrenic"[Title/Abstract]) OR (“eating disorder”[Title/Abstract]) OR (“anorexia”[Title/Abstract]) OR (“bulimia”[Title/Abstract]) OR (substance-related disorders[MeSH Terms]) OR

		(“addiction”[Title/Abstract]) OR (“addicted”[Title/Abstract]) OR (“overdose”[Title/Abstract]) OR (“substance abuse”[Title/Abstract]) OR (“alcohol abuse”[Title/Abstract]) OR (“drug abuse”[Title/Abstract]) OR (“intoxication”[Title/Abstract]) OR (“intoxicated”[Title/Abstract])) **AND** ((emergency nursing[MeSH Terms]) OR (emergency medicine[MeSH Terms]) OR (psychology[MeSH Terms]) OR (psychiatry[MeSH Terms]) OR (patients[MeSH Terms]) OR (family[MeSH Terms]) OR (parents[MeSH Terms]) OR (caregivers[MeSH Terms]) OR ("patient"[Title/Abstract]) OR ("doctor"[Title/Abstract]) OR ("physician"[Title/Abstract]) OR ("nurse"[Title/Abstract]) OR ("clinician"[Title/Abstract]) OR ("healthcare worker"[Title/Abstract]) OR ("psychiatrist"[Title/Abstract]) OR ("psychologist"[Title/Abstract]) OR ("social worker"[Title/Abstract]) OR ("counselor"[Title/Abstract]) OR ("therapist"[Title/Abstract]) OR ("care team"[Title/Abstract]) OR ("security"[Title/Abstract]) OR ("ems"[Title/Abstract]) OR (“family”[Title/Abstract]) OR (“parents”[Title/Abstract]) OR (“caregivers”[Title/Abstract])))
	Intervention	**AND** (("built environment"[MeSH Terms]) OR (hospital design and construction[MeSH Terms]) OR ("built environment"[Title/Abstract]) OR ("physical environment"[Title/Abstract]) OR ("architecture"[Title/Abstract]) OR ("interior design"[Title/Abstract]) OR ("environmental design"[Title/Abstract]) OR ("lighting"[Title/Abstract]) OR ("daylight"[Title/Abstract]) OR ("window"[Title/Abstract]) OR ("crisis stabilization"[Title/Abstract]) OR ("layout"[Title/Abstract]) OR ("visibility"[Title/Abstract]) OR ("furniture"[Title/Abstract]) OR ("decoration"[Title/Abstract]) OR ("decor"[Title/Abstract]) OR ("art"[Title/Abstract]) OR ("positive distraction"[Title/Abstract]) OR ("noise"[Title/Abstract]) OR ("ergonomics"[Title/Abstract]) OR ("ergonomic"[Title/Abstract]) OR ("odor"[Title/Abstract]) OR ("smell"[Title/Abstract]) OR ("anti ligature"[Title/Abstract]) OR ("ligature resistant"[Title/Abstract]) OR ("sensory room"[Title/Abstract]) OR ("sensory friendly"[Title/Abstract]) OR ("snoezelen"[Title/Abstract]))
	Outcome	**AND** ((defense mechanisms[MeSH Terms]) OR (emotions[MeSH Terms]) OR (behavioral symptoms[MeSH Terms]) OR (length of stay[MeSH Terms]) OR (patient participation[MeSH Terms]) OR (family relationships, professional[MeSH Terms]) OR ("aggression"[Title/Abstract]) OR ("aggressive"[Title/Abstract]) OR ("agitation"[Title/Abstract]) OR ("agitated"[Title/Abstract]) OR ("confusion"[Title/Abstract]) OR ("distress"[Title/Abstract]) OR ("anger"[Title/Abstract]) OR ("angry"[Title/Abstract]) OR ("comfort"[Title/Abstract]) OR ("discomfort"[Title/Abstract]) OR ("pain"[Title/Abstract]) OR ("satisfaction"[Title/Abstract]) OR ("dissatisfaction"[Title/Abstract]) OR ("privacy"[Title/Abstract]) OR ("private"[Title/Abstract]) OR ("safe"[Title/Abstract]) OR ("safety"[Title/Abstract]) OR ("secure"[Title/Abstract]) OR ("security"[Title/Abstract]) OR ("violent"[Title/Abstract]) OR ("violence"[Title/Abstract]) OR ("anxiety"[Title/Abstract]) OR ("anxious"[Title/Abstract]) OR ("stress"[Title/Abstract]) OR ("strain"[Title/Abstract]) OR ("self harm"[Title/Abstract]) OR ("self injury"[Title/Abstract]) OR ("suicide"[Title/Abstract]) OR ("suicidal"[Title/Abstract]) OR ("sedation"[Title/Abstract]) OR ("sedate"[Title/Abstract]) OR ("sedative"[Title/Abstract]) OR ("restrain"[Title/Abstract]) OR ("restraint"[Title/Abstract]) OR ("ligature"[Title/Abstract]) OR ("risk"[Title/Abstract]) OR ("resilience"[Title/Abstract]) OR ("length of stay"[Title/Abstract]) OR ("leaving without being seen"[Title/Abstract]) OR ("lwbs"[Title/Abstract]) OR ("elope"[Title/Abstract]) OR ("elopement"[Title/Abstract]) OR ("injure"[Title/Abstract]) OR

		("injury"[Title/Abstract]) OR ("family engagement"[Title/Abstract]) OR ("transfer"[Title/Abstract]) OR (“wellbeing"[Title/Abstract]) OR (“outpatient follow up"[Title/Abstract]) OR (“ambulatory follow up"[Title/Abstract]))
C) Tools & Technology	Setting	((emergency service, hospital[MeSH Terms]) OR (emergency services, psychiatric[MeSH Terms]) OR ("emergency department"[Title/Abstract]) OR ("emergency room"[Title/Abstract]) OR ("emergency service"[Title/Abstract]) OR ("emergency ward"[Title/Abstract]) OR ("emergency clinic"[Title/Abstract]) OR ("emergency unit"[Title/Abstract]) OR ("accident and emergency"[Title/Abstract]))
	Population	**AND** ((("mental health"[MeSH Terms]) OR (behavioral medicine[MeSH Terms]) OR (mental disorders[MeSH Terms]) OR (behavioral medicine[MeSH Terms]) OR ("mental health"[Title/Abstract]) OR ("behavioral health"[Title/Abstract]) OR ("psychiatry"[Title/Abstract]) OR ("psychology"[Title/Abstract]) OR ("psychiatric"[Title/Abstract]) OR ("autism"[Title/Abstract]) OR ("autism spectrum"[Title/Abstract]) OR ("cognitive impairment"[Title/Abstract]) OR ("intellectual impairment"[Title/Abstract]) OR ("cognitively impaired"[Title/Abstract]) OR ("intellectually impaired"[Title/Abstract]) OR ("depression"[Title/Abstract]) OR ("anxiety"[Title/Abstract]) OR ("attention deficit"[Title/Abstract]) OR ("hyperactivity"[Title/Abstract]) OR ("adhd"[Title/Abstract]) OR ("post traumatic stress"[Title/Abstract]) OR ("ptsd"[Title/Abstract]) OR ("obsessive compulsive"[Title/Abstract]) OR ("tourette"[Title/Abstract]) OR ("oppositional defiant"[Title/Abstract]) OR ("mania"[Title/Abstract]) OR ("manic"[Title/Abstract]) OR ("depressive"[Title/Abstract]) OR ("suicidal"[Title/Abstract]) OR ("schizophrenia"[Title/Abstract]) OR ("schizophrenic"[Title/Abstract]) OR (“eating disorder”[Title/Abstract]) OR (“anorexia”[Title/Abstract]) OR (“bulimia”[Title/Abstract]) OR (substance-related disorders[MeSH Terms]) OR (“addiction”[Title/Abstract]) OR (“addicted”[Title/Abstract]) OR (“overdose”[Title/Abstract]) OR (“substance abuse”[Title/Abstract]) OR (“alcohol abuse”[Title/Abstract]) OR (“drug abuse”[Title/Abstract]) OR (“intoxication”[Title/Abstract]) OR (“intoxicated”[Title/Abstract])) **AND** ((emergency nursing[MeSH Terms]) OR (emergency medicine[MeSH Terms]) OR (psychology[MeSH Terms]) OR (psychiatry[MeSH Terms]) OR (patients[MeSH Terms]) OR (family[MeSH Terms]) OR (parents[MeSH Terms) OR (caregivers[MeSH Terms]) OR ("patient"[Title/Abstract]) OR ("doctor"[Title/Abstract]) OR ("physician"[Title/Abstract]) OR ("nurse"[Title/Abstract]) OR ("clinician"[Title/Abstract]) OR ("healthcare worker"[Title/Abstract]) OR ("psychiatrist"[Title/Abstract]) OR ("psychologist"[Title/Abstract]) OR ("social worker"[Title/Abstract]) OR ("counselor"[Title/Abstract]) OR ("therapist"[Title/Abstract]) OR ("care team"[Title/Abstract]) OR ("security"[Title/Abstract]) OR ("ems"[Title/Abstract]) OR (“family”[Title/Abstract]) OR (“parents”[Title/Abstract]) OR (“caregivers”[Title/Abstract])))
	Intervention	**AND** ((technology[MeSH Terms]) OR (telemedicine[MeSH Terms]) OR ("email"[Title/Abstract]) OR ("electronic mail"[Title/Abstract]) OR ("internet"[Title/Abstract]) OR ("computer"[Title/Abstract]) OR ("medical informatics"[Title/Abstract]) OR ("software"[Title/Abstract]) OR ("telephone"[Title/Abstract]) OR ("technology"[Title/Abstract]) OR ("kiosk"[Title/Abstract]) OR ("mobile app"[Title/Abstract]) OR ("mobile application"[Title/Abstract]) OR ("wireless"[Title/Abstract]) OR ("mobile"[Title/Abstract]) OR ("text message"[Title/Abstract]) OR ("message"[Title/Abstract]) OR ("messaging"[Title/Abstract]) OR ("sms"[Title/Abstract]) OR ("short messaging service"[Title/Abstract]) OR ("mhealth"[Title/Abstract]) OR ("ehealth"[Title/Abstract]) OR ("handheld"[Title/Abstract]) OR ("laptop"[Title/Abstract]) OR ("palmtop"[Title/Abstract]) OR ("tablet"[Title/Abstract]) OR

		("smartphone"[Title/Abstract]) OR ("social network"[Title/Abstract]) OR ("electronic health record"[Title/Abstract]) OR ("electronic medical record"[Title/Abstract]) OR ("ehr"[Title/Abstract]) OR ("emr"[Title/Abstract]) OR ("telehealth"[Title/Abstract]) OR ("telepsychiatry"[Title/Abstract]) OR ("telepsychology"[Title/Abstract]) OR ("simulation"[Title/Abstract]))
	Outcome	**AND** ((defense mechanisms[MeSH Terms]) OR (emotions[MeSH Terms]) OR (behavioral symptoms[MeSH Terms]) OR (length of stay[MeSH Terms]) OR (patient participation[MeSH Terms]) OR (family relationships, professional[MeSH Terms]) OR ("aggression"[Title/Abstract]) OR ("aggressive"[Title/Abstract]) OR ("agitation"[Title/Abstract]) OR ("agitated"[Title/Abstract]) OR ("confusion"[Title/Abstract]) OR ("distress"[Title/Abstract]) OR ("anger"[Title/Abstract]) OR ("angry"[Title/Abstract]) OR ("comfort"[Title/Abstract]) OR ("discomfort"[Title/Abstract]) OR ("pain"[Title/Abstract]) OR ("satisfaction"[Title/Abstract]) OR ("dissatisfaction"[Title/Abstract]) OR ("privacy"[Title/Abstract]) OR ("private"[Title/Abstract]) OR ("safe"[Title/Abstract]) OR ("safety"[Title/Abstract]) OR ("secure"[Title/Abstract]) OR ("security"[Title/Abstract]) OR ("violent"[Title/Abstract]) OR ("violence"[Title/Abstract]) OR ("anxiety"[Title/Abstract]) OR ("anxious"[Title/Abstract]) OR ("stress"[Title/Abstract]) OR ("strain"[Title/Abstract]) OR ("self harm"[Title/Abstract]) OR ("self injury"[Title/Abstract]) OR ("suicide"[Title/Abstract]) OR ("suicidal"[Title/Abstract]) OR ("sedation"[Title/Abstract]) OR ("sedate"[Title/Abstract]) OR ("sedative"[Title/Abstract]) OR ("restrain"[Title/Abstract]) OR ("restraint"[Title/Abstract]) OR ("ligature"[Title/Abstract]) OR ("risk"[Title/Abstract]) OR ("resilience"[Title/Abstract]) OR ("length of stay"[Title/Abstract]) OR ("leaving without being seen"[Title/Abstract]) OR ("lwbs"[Title/Abstract]) OR ("elope"[Title/Abstract]) OR ("elopement"[Title/Abstract]) OR ("injure"[Title/Abstract]) OR ("injury"[Title/Abstract]) OR ("family engagement"[Title/Abstract]) OR ("transfer"[Title/Abstract]) OR ("wellbeing"[Title/Abstract]) OR ("outpatient follow up"[Title/Abstract]) OR ("ambulatory follow up"[Title/Abstract]))
CINAHL		
A) Tasks & Organizational Conditions	Setting	((MH "emergency service") OR (MH "emergency services, psychiatric") OR (TI "emergency department" OR AB "emergency department") OR (TI "emergency room" OR AB "emergency room") OR (TI "emergency service" OR AB "emergency service") OR (TI "emergency ward" OR AB "emergency ward") OR (TI "emergency clinic" OR AB "emergency clinic") OR (TI "emergency unit" OR AB "emergency unit") OR (TI "accident and emergency" OR AB "accident and emergency"))
	Population	**AND** (((MH "mental health") OR (MH "behavioral and mental disorders") OR (TI "mental health" OR AB "mental health") OR (TI "behavioral health" OR AB "behavioral health") OR (TI psychiatry OR AB psychiatry) OR (TI psychology OR AB psychology) OR (TI psychiatric OR AB psychiatric) OR (TI autism OR AB autism) OR (TI "autism spectrum" OR AB "autism spectrum") OR (TI "cognitive impairment" OR AB "cognitive impairment") OR (TI "intellectual impairment" OR AB "intellectual impairment") OR (TI "cognitively impaired" OR AB "cognitively impaired") OR (TI "intellectually impaired" OR AB "intellectually impaired") OR (TI depression OR AB depression) OR (TI anxiety OR AB anxiety) OR (TI "attention deficit" OR AB "attention deficit") OR (TI hyperactivity OR AB hyperactivity) OR (TI adhd OR AB adhd) OR (TI "post traumatic stress" OR AB "post traumatic stress") OR (TI ptsd OR AB ptsd) OR (TI "obsessive compulsive" OR AB "obsessive compulsive") OR (TI tourette OR AB tourette) OR (TI "oppositional defiant" OR AB "oppositional defiant") OR (TI mania OR AB mania) OR (TI manic OR AB manic) OR (TI depressive OR

		AB depressive) OR (TI suicidal OR AB suicidal) OR (TI schizophrenia OR AB schizophrenia) OR (TI "schizophrenic" OR AB "schizophrenic") OR (TI "eating disorder" OR AB "eating disorder") OR (TI "anorexia" OR AB "anorexia") OR (TI "bulimia" OR AB "bulimia") OR (MH "substance abuse") OR (TI addiction OR AB addiction) OR (TI addicted OR AB addicted) OR (TI overdose OR AB overdose) OR (TI "substance abuse" OR AB "substance abuse") OR (TI "drug abuse" OR AB "drug abuse") OR (TI "alcohol abuse" OR AB "alcohol abuse") OR (TI intoxicated OR AB intoxicated) OR (TI intoxication OR AB intoxication)) **AND** ((MH "emergency nursing") OR (MH "emergency medicine") OR (MH psychology) OR (MH psychiatry) OR (MH "mental health personnel") OR (MH patients) OR (MH "psychiatric patient") OR (MH family) OR (MH parents) OR (MH caregivers) OR (TI patient OR AB patient) OR (TI family OR AB family) OR (TI parent OR AB parent) OR (TI caregiver OR AB caregiver) OR (TI doctor OR AB doctor) OR (TI physician OR AB physician) OR (TI nurse OR AB nurse) OR (TI clinician OR AB clinician) OR (TI "healthcare worker" OR AB "healthcare worker") OR (TI psychiatrist OR AB psychiatrist) OR (TI psychologist OR AB psychologist) OR (TI "social worker" OR AB "social worker") OR (TI counselor OR AB counselor) OR (TI therapist OR AB therapist) OR (TI "care team" OR AB "care team") OR (TI security OR AB security) OR (TI ems OR AB ems)))
	Intervention	**AND** ((MH "education, medical, continuing") OR (MH "education, nursing, continuing") OR (MH "education, emergency medical services") OR (MH workflow) OR (MH "decision making, clinical") OR (TI task OR AB task) OR (TI process OR AB process) OR (TI workflow OR AB workflow) OR (TI triage OR AB triage) OR (TI "fast track" OR AB "fast track") OR (TI lean OR AB lean) OR (TI treatment OR AB treatment) OR (TI assess OR AB assess) OR (TI assessment OR AB assessment) OR (TI screening OR AB screening) OR (TI "medical screening" OR AB "medical screening") OR (TI "psychiatric screening" OR AB "psychiatric screening") OR (TI evaluation OR AB evaluation) OR (TI evaluate OR AB evaluate) OR (TI training OR AB training) OR (TI "professional development" OR AB "professional development") OR (TI mentor OR AB mentor) OR (TI mentorship OR AB mentorship) OR (TI learning OR AB learning) OR (TI education OR AB education) OR (TI culture OR AB culture) OR (TI management OR AB management) OR (TI manager OR AB manager) OR (TI change OR AB change) OR (TI leader OR AB leader) OR (TI leadership OR AB leadership) OR (TI team OR AB team) OR (TI teamwork OR AB teamwork) OR (TI policy OR AB policy) OR (TI policies OR AB policies) OR (TI staffing OR AB staffing) OR (TI scheduling OR AB scheduling) OR (TI schedule OR AB schedule))
	Outcome	**AND** ((MH "defense mechanisms") OR (MH emotions) OR (MH "behavioral symptoms") OR (MH "length of stay") OR (MH "professional-patient relations") OR (MH "professional-family relations") OR (TI aggression OR AB aggression) OR (TI aggressive OR AB aggressive) OR (TI agitation OR AB agitation) OR (TI agitated OR AB agitated) OR (TI confusion OR AB confusion) OR (TI distress OR AB distress) OR (TI anger OR AB anger) OR (TI angry OR AB angry) OR (TI comfort OR AB comfort) OR (TI discomfort OR AB discomfort) OR (TI pain OR AB pain) OR (TI satisfaction OR AB satisfaction) OR (TI dissatisfaction OR AB dissatisfaction) OR (TI privacy OR AB privacy) OR (TI private OR AB private) OR (TI safe OR AB safe) OR (TI safety OR AB safety) OR (TI secure OR AB secure) OR (TI security OR AB security) OR (TI violent OR AB violent) OR (TI violence OR AB violence) OR (TI anxiety OR AB anxiety) OR (TI anxious OR AB anxious) OR (TI stress OR AB stress) OR (TI strain OR AB strain) OR (TI "self harm" OR AB "self harm") OR (TI "self injury" OR AB "self injury") OR (TI suicide OR AB suicide) OR (TI suicidal OR AB suicidal) OR (TI sedation OR AB sedation) OR (TI sedate OR AB sedate) OR (TI sedative OR AB sedative) OR (TI restrain OR AB restrain) OR (TI

		restraint OR AB restraint) OR (TI ligature OR AB ligature) OR (TI risk OR AB risk) OR (TI resilience OR AB resilience) OR (TI "length of stay" OR AB "length of stay") OR (TI "leaving without being seen" OR AB "leaving without being seen") OR (TI lwbs OR AB lwbs) OR (TI elope OR AB elope) OR (TI elopement OR AB elopement) OR (TI injure OR AB injure) OR (TI injury OR AB injury) OR (TI "family engagement" OR AB "family engagement") OR (TI transfer OR AB transfer) OR (TI wellbeing OR AB wellbeing) OR (TI "outpatient follow up" OR AB "outpatient follow up") OR (TI "ambulatory follow up" OR AB "ambulatory follow up"))
B) Built Environment	Setting	((MH "emergency service") OR (MH "emergency services, psychiatric") OR (TI "emergency department" OR AB "emergency department") OR (TI "emergency room" OR AB "emergency room") OR (TI "emergency service" OR AB "emergency service") OR (TI "emergency ward" OR AB "emergency ward") OR (TI "emergency clinic" OR AB "emergency clinic") OR (TI "emergency unit" OR AB "emergency unit") OR (TI "accident and emergency" OR AB "accident and emergency"))
	Population	**AND** (((MH "mental health") OR (MH "behavioral and mental disorders") OR (TI "mental health" OR AB "mental health") OR (TI "behavioral health" OR AB "behavioral health") OR (TI psychiatry OR AB psychiatry) OR (TI psychology OR AB psychology) OR (TI psychiatric OR AB psychiatric) OR (TI autism OR AB autism) OR (TI "autism spectrum" OR AB "autism spectrum") OR (TI "cognitive impairment" OR AB "cognitive impairment") OR (TI "intellectual impairment" OR AB "intellectual impairment") OR (TI "cognitively impaired" OR AB "cognitively impaired") OR (TI "intellectually impaired" OR AB "intellectually impaired") OR (TI depression OR AB depression) OR (TI anxiety OR AB anxiety) OR (TI "attention deficit" OR AB "attention deficit") OR (TI hyperactivity OR AB hyperactivity) OR (TI adhd OR AB adhd) OR (TI "post traumatic stress" OR AB "post traumatic stress") OR (TI ptsd OR AB ptsd) OR (TI "obsessive compulsive" OR AB "obsessive compulsive") OR (TI tourette OR AB tourette) OR (TI "oppositional defiant" OR AB "oppositional defiant") OR (TI mania OR AB mania) OR (TI manic OR AB manic) OR (TI depressive OR AB depressive) OR (TI suicidal OR AB suicidal) OR (TI schizophrenia OR AB schizophrenia) OR (TI "schizophrenic" OR AB "schizophrenic") OR (TI "eating disorder" OR AB "eating disorder") OR (TI "anorexia" OR AB "anorexia") OR (TI "bulimia" OR AB "bulimia") OR (MH "substance abuse") OR (TI addiction OR AB addiction) OR (TI addicted OR AB addicted) OR (TI overdose OR AB overdose) OR (TI "substance abuse" OR AB "substance abuse") OR (TI "drug abuse" OR AB "drug abuse") OR (TI "alcohol abuse" OR AB "alcohol abuse") OR (TI intoxicated OR AB intoxicated) OR (TI intoxication OR AB intoxication)) **AND** ((MH "emergency nursing") OR (MH "emergency medicine") OR (MH psychology) OR (MH psychiatry) OR (MH "mental health personnel") OR (MH patients) OR (MH "psychiatric patient") OR (MH family) OR (MH parents) OR (MH caregivers) OR (TI patient OR AB patient) OR (TI family OR AB family) OR (TI parent OR AB parent) OR (TI caregiver OR AB caregiver) OR (TI doctor OR AB doctor) OR (TI physician OR AB physician) OR (TI nurse OR AB nurse) OR (TI clinician OR AB clinician) OR (TI "healthcare worker" OR AB "healthcare worker") OR (TI psychiatrist OR AB psychiatrist) OR (TI psychologist OR AB psychologist) OR (TI "social worker" OR AB "social worker") OR (TI counselor OR AB counselor) OR (TI therapist OR AB therapist) OR (TI "care team" OR AB "care team") OR (TI security OR AB security) OR (TI ems OR AB ems)))
	Intervention	**AND** ((MH "built environment") OR (MH "hospital design and construction") OR (TI "built environment" OR AB "built environment") OR (TI "physical environment" OR AB "physical environment") OR (TI architecture OR AB architecture) OR (TI "interior design" OR AB "interior design") OR (TI lighting OR AB lighting) OR (TI daylight OR AB daylight) OR (TI window OR AB

		window) OR (TI “crisis stabilization” OR AB “crisis stabilization”) OR (TI layout OR AB layout) OR (TI visibility OR AB visibility) OR (TI furniture OR AB furniture) OR (TI decoration OR AB decoration) OR (TI decor OR AB decor) OR (TI art OR AB art) OR (TI “positive distraction” OR AB “positive distraction”) OR (TI noise OR AB noise) OR (TI ergonomics OR AB ergonomics) OR (TI ergonomic OR AB ergonomic) OR (TI odor OR AB odor) OR (TI smell OR AB smell) OR (TI “anti ligature” OR AB “anti ligature”) OR (TI “ligature resistant” OR AB “ligature resistant”) OR (TI “sensory room” OR AB “sensory room”) OR (TI “snoezelen” OR AB “snoezelen”))
	Outcome	**AND** ((MH "defense mechanisms") OR (MH emotions) OR (MH "behavioral symptoms") OR (MH "length of stay") OR (MH “professional-patient relations”) OR (MH “professional-family relations”) OR (TI aggression OR AB aggression) OR (TI aggressive OR AB aggressive) OR (TI agitation OR AB agitation) OR (TI agitated OR AB agitated) OR (TI confusion OR AB confusion) OR (TI distress OR AB distress) OR (TI anger OR AB anger) OR (TI angry OR AB angry) OR (TI comfort OR AB comfort) OR (TI discomfort OR AB discomfort) OR (TI pain OR AB pain) OR (TI satisfaction OR AB satisfaction) OR (TI dissatisfaction OR AB dissatisfaction) OR (TI privacy OR AB privacy) OR (TI private OR AB private) OR (TI safe OR AB safe) OR (TI safety OR AB safety) OR (TI secure OR AB secure) OR (TI security OR AB security) OR (TI violent OR AB violent) OR (TI violence OR AB violence) OR (TI anxiety OR AB anxiety) OR (TI anxious OR AB anxious) OR (TI stress OR AB stress) OR (TI strain OR AB strain) OR (TI "self harm" OR AB "self harm") OR (TI "self injury" OR AB "self injury") OR (TI suicide OR AB suicide) OR (TI suicidal OR AB suicidal) OR (TI sedation OR AB sedation) OR (TI sedate OR AB sedate) OR (TI sedative OR AB sedative) OR (TI restrain OR AB restrain) OR (TI restraint OR AB restraint) OR (TI ligature OR AB ligature) OR (TI risk OR AB risk) OR (TI resilience OR AB resilience) OR (TI "length of stay" OR AB "length of stay") OR (TI "leaving without being seen" OR AB "leaving without being seen") OR (TI lwbs OR AB lwbs) OR (TI elope OR AB elope) OR (TI elopement OR AB elopement) OR (TI injure OR AB injure) OR (TI injury OR AB injury) OR (TI "family engagement" OR AB "family engagement") OR (TI transfer OR AB transfer) OR (TI wellbeing OR AB wellbeing) OR (TI “outpatient follow up” OR AB “outpatient follow up”) OR (TI “ambulatory follow up” OR AB “ambulatory follow up”))
C) Tools & Technology	Setting	((MH "emergency service") OR (MH "emergency services, psychiatric") OR (TI "emergency department" OR AB "emergency department") OR (TI "emergency room" OR AB "emergency room") OR (TI "emergency service" OR AB "emergency service") OR (TI "emergency ward" OR AB "emergency ward") OR (TI "emergency clinic" OR AB "emergency clinic") OR (TI "emergency unit" OR AB "emergency unit") OR (TI "accident and emergency" OR AB "accident and emergency"))
	Population	**AND** (((MH "mental health") OR (MH "behavioral and mental disorders") OR (TI "mental health" OR AB "mental health") OR (TI "behavioral health" OR AB "behavioral health") OR (TI psychiatry OR AB psychiatry) OR (TI psychology OR AB psychology) OR (TI psychiatric OR AB psychiatric) OR (TI autism OR AB autism) OR (TI "autism spectrum" OR AB "autism spectrum") OR (TI "cognitive impairment" OR AB "cognitive impairment") OR (TI "intellectual impairment" OR AB "intellectual impairment") OR (TI "cognitively impaired" OR AB "cognitively impaired") OR (TI "intellectually impaired" OR AB "intellectually impaired") OR (TI depression OR AB depression) OR (TI anxiety OR AB anxiety) OR (TI "attention deficit" OR AB "attention deficit") OR (TI hyperactivity OR AB hyperactivity) OR (TI adhd OR AB adhd) OR (TI "post traumatic stress" OR AB "post traumatic stress") OR (TI ptsd OR AB ptsd) OR (TI "obsessive compulsive" OR AB "obsessive compulsive") OR (TI tourette OR AB tourette) OR (TI "oppositional defiant" OR AB "oppositional defiant") OR

		(TI mania OR AB mania) OR (TI manic OR AB manic) OR (TI depressive OR AB depressive) OR (TI suicidal OR AB suicidal) OR (TI schizophrenia OR AB schizophrenia) OR (TI "schizophrenic" OR AB “schizophrenic”) OR (TI “eating disorder” OR AB “eating disorder”) OR (TI “anorexia” OR AB “anorexia”) OR (TI “bulimia” OR AB “bulimia”) OR (MH “substance abuse”) OR (TI addiction OR AB addiction) OR (TI addicted OR AB addicted) OR (TI overdose OR AB overdose) OR (TI “substance abuse” OR AB “substance abuse”) OR (TI “drug abuse” OR AB “drug abuse”) OR (TI “alcohol abuse” OR AB “alcohol abuse”) OR (TI intoxicated OR AB intoxicated) OR (TI intoxication OR AB intoxication)) **AND** ((MH "emergency nursing") OR (MH "emergency medicine") OR (MH psychology) OR (MH psychiatry) OR (MH “mental health personnel”) OR (MH patients) OR (MH “psychiatric patient”) OR (MH family) OR (MH parents) OR (MH caregivers) OR (TI patient OR AB patient) OR (TI family OR AB family) OR (TI parent OR AB parent) OR (TI caregiver OR AB caregiver) OR (TI doctor OR AB doctor) OR (TI physician OR AB physician) OR (TI nurse OR AB nurse) OR (TI clinician OR AB clinician) OR (TI "healthcare worker" OR AB "healthcare worker") OR (TI psychiatrist OR AB psychiatrist) OR (TI psychologist OR AB psychologist) OR (TI "social worker" OR AB "social worker") OR (TI counselor OR AB counselor) OR (TI therapist OR AB therapist) OR (TI "care team" OR AB "care team") OR (TI security OR AB security) OR (TI ems OR AB ems)))
	Intervention	**AND** ((MH technology) OR (MH telemedicine) OR (TI email OR AB email) OR (TI “electronic mail” OR AB “electronic mail”) OR (TI internet OR AB internet) OR (TI computer OR AB computer) OR (TI “medical informatics” OR AB “medical informatics”) OR (TI software OR AB software) OR (TI telephone OR AB telephone) OR (TI technology OR AB technology) OR (TI kiosk OR AB kiosk) OR (TI “mobile app” OR AB “mobile app”) OR (TI “mobile application” OR AB “mobile application”) OR (TI wireless OR AB wireless) OR (TI mobile OR AB mobile) OR (TI “text message” OR AB “text message”) OR (TI message OR AB message) OR (TI messaging OR AB messaging) OR (TI sms OR AB sms) OR (TI “short messaging service” OR AB “short messaging service”) OR (TI mhealth OR AB mhealth) OR (TI ehealth OR AB ehealth) OR (TI handheld OR AB handheld) OR (TI laptop OR AB laptop) OR (TI palmtop OR AB palmtop) OR (TI tablet OR AB tablet) OR (TI smartphone OR AB smartphone) OR (TI “social network” OR AB “social network”) OR (TI “electronic health record” OR AB “electronic health record”) OR (TI “electronic medical record” OR AB “electronic medical record”) OR (TI ehr OR AB ehr) OR (TI emr OR AB emr) OR (TI telehealth OR AB telehealth) OR (TI telepsychiatry OR AB telepsychiatry) OR (TI telepsychology OR AB telepsychology) OR (TI simulation OR AB simulation))
	Outcome	**AND** ((MH "defense mechanisms") OR (MH emotions) OR (MH "behavioral symptoms") OR (MH "length of stay") OR (MH “professional-patient relations”) OR (MH “professional-family relations”) OR (TI aggression OR AB aggression) OR (TI aggressive OR AB aggressive) OR (TI agitation OR AB agitation) OR (TI agitated OR AB agitated) OR (TI confusion OR AB confusion) OR (TI distress OR AB distress) OR (TI anger OR AB anger) OR (TI angry OR AB angry) OR (TI comfort OR AB comfort) OR (TI discomfort OR AB discomfort) OR (TI pain OR AB pain) OR (TI satisfaction OR AB satisfaction) OR (TI dissatisfaction OR AB dissatisfaction) OR (TI privacy OR AB privacy) OR (TI private OR AB private) OR (TI safe OR AB safe) OR (TI safety OR AB safety) OR (TI secure OR AB secure) OR (TI security OR AB security) OR (TI violent OR AB violent) OR (TI violence OR AB violence) OR (TI anxiety OR AB anxiety) OR (TI anxious OR AB anxious) OR (TI stress OR AB stress) OR (TI strain OR AB strain) OR (TI "self harm" OR AB "self harm") OR (TI "self injury" OR AB "self injury") OR (TI suicide OR AB suicide) OR (TI suicidal OR AB suicidal) OR (TI sedation OR AB sedation) OR (TI sedate OR AB sedate)

		OR (TI sedative OR AB sedative) OR (TI restrain OR AB restrain) OR (TI restraint OR AB restraint) OR (TI ligature OR AB ligature) OR (TI risk OR AB risk) OR (TI resilience OR AB resilience) OR (TI "length of stay" OR AB "length of stay") OR (TI "leaving without being seen" OR AB "leaving without being seen") OR (TI lwbs OR AB lwbs) OR (TI elope OR AB elope) OR (TI elopement OR AB elopement) OR (TI injure OR AB injure) OR (TI injury OR AB injury) OR (TI "family engagement" OR AB "family engagement") OR (TI transfer OR AB transfer) OR (TI wellbeing OR AB wellbeing) OR (TI “outpatient follow up” OR AB “outpatient follow up”) OR (TI “ambulatory follow up” OR AB “ambulatory follow up”))
Medline		
A) Tasks & Organizational Conditions	Setting	((MH "emergency service, hospital") OR (MH "emergency services, psychiatric") OR (TI "emergency department" OR AB "emergency department") OR (TI "emergency room" OR AB "emergency room") OR (TI "emergency service" OR AB "emergency service") OR (TI "emergency ward" OR AB "emergency ward") OR (TI "emergency clinic" OR AB "emergency clinic") OR (TI "emergency unit" OR AB "emergency unit") OR (TI "accident and emergency" OR AB "accident and emergency"))
	Population	**AND** (((MH "mental health") OR (MH "behavioral medicine") OR (MH “mental disorders”) OR (TI "mental health" OR AB "mental health") OR (TI "behavioral health" OR AB "behavioral health") OR (TI psychiatry OR AB psychiatry) OR (TI psychology OR AB psychology) OR (TI psychiatric OR AB psychiatric) OR (TI autism OR AB autism) OR (TI "autism spectrum" OR AB "autism spectrum") OR (TI "cognitive impairment" OR AB "cognitive impairment") OR (TI "intellectual impairment" OR AB "intellectual impairment") OR (TI "cognitively impaired" OR AB "cognitively impaired") OR (TI "intellectually impaired" OR AB "intellectually impaired") OR (TI depression OR AB depression) OR (TI anxiety OR AB anxiety) OR (TI "attention deficit" OR AB "attention deficit") OR (TI hyperactivity OR AB hyperactivity) OR (TI adhd OR AB adhd) OR (TI "post traumatic stress" OR AB "post traumatic stress") OR (TI ptsd OR AB ptsd) OR (TI "obsessive compulsive" OR AB "obsessive compulsive") OR (TI tourette OR AB tourette) OR (TI "oppositional defiant" OR AB "oppositional defiant") OR (TI mania OR AB mania) OR (TI manic OR AB manic) OR (TI depressive OR AB depressive) OR (TI suicidal OR AB suicidal) OR (TI schizophrenia OR AB schizophrenia) OR (TI "schizophrenic" OR AB “schizophrenic”) OR (TI “eating disorder” OR AB “eating disorder”) OR (TI “anorexia” OR AB “anorexia”) OR (TI “bulimia” OR AB “bulimia”) OR (MH “substance-related disorders”) OR (TI addiction OR AB addiction) OR (TI addicted OR AB addicted) OR (TI overdose OR AB overdose) OR (TI “substance abuse” OR AB “substance abuse”) OR (TI “drug abuse” OR AB “drug abuse”) OR (TI “alcohol abuse” OR AB “alcohol abuse”) OR (TI intoxicated OR AB intoxicated) OR (TI intoxication OR AB intoxication)) **AND** ((MH "emergency nursing") OR (MH "emergency medicine") OR (MH “psychology”) OR (MH “psychiatry”) OR (MH “patients”) OR (MH “family”) OR (MH “parents”) OR (MH “caregivers”) OR (TI patient OR AB patient) OR (TI family OR AB family) OR (TI parent OR AB parent) OR (TI caregiver OR AB caregiver) OR (TI doctor OR AB doctor) OR (TI physician OR AB physician) OR (TI nurse OR AB nurse) OR (TI clinician OR AB clinician) OR (TI "healthcare worker" OR AB "healthcare worker") OR (TI psychiatrist OR AB psychiatrist) OR (TI psychologist OR AB psychologist) OR (TI "social worker" OR AB "social worker") OR (TI counselor OR AB counselor) OR (TI therapist OR AB therapist) OR (TI "care team" OR AB "care team") OR (TI security OR AB security) OR (TI ems OR AB ems)))
	Intervention	**AND** ((MH “education, continuing”) OR (MH “education, medical”) OR (MH “education, nursing”) OR (MH “workflow”) OR (MH “decision making, clinical”) OR (TI task OR AB task) OR (TI process OR AB process) OR (TI workflow OR AB workflow) OR (TI triage OR AB triage) OR (TI "fast track"

		OR AB "fast track") OR (TI lean OR AB lean) OR (TI treatment OR AB treatment) OR (TI assess OR AB assess) OR (TI assessment OR AB assessment) OR (TI screening OR AB screening) OR (TI "medical screening" OR AB "medical screening") OR (TI "psychiatric screening" OR AB "psychiatric screening") OR (TI evaluation OR AB evaluation) OR (TI evaluate OR AB evaluate) OR (TI training OR AB training) OR (TI "professional development" OR AB "professional development") OR (TI mentor OR AB mentor) OR (TI mentorship OR AB mentorship) OR (TI learning OR AB learning) OR (TI education OR AB education) OR (TI culture OR AB culture) OR (TI management OR AB management) OR (TI manager OR AB manager) OR (TI change OR AB change) OR (TI leader OR AB leader) OR (TI leadership OR AB leadership) OR (TI team OR AB team) OR (TI teamwork OR AB teamwork) OR (TI policy OR AB policy) OR (TI policies OR AB policies) OR (TI staffing OR AB staffing) OR (TI scheduling OR AB scheduling) OR (TI schedule OR AB schedule))
	Outcome	**AND** ((MH "defense mechanisms") OR (MH "emotions") OR (MH "behavioral symptoms") OR (MH "length of stay") OR (MH "patient participation") OR (MH "family relationships, professional") OR (TI aggression OR AB aggression) OR (TI aggressive OR AB aggressive) OR (TI agitation OR AB agitation) OR (TI agitated OR AB agitated) OR (TI confusion OR AB confusion) OR (TI distress OR AB distress) OR (TI anger OR AB anger) OR (TI angry OR AB angry) OR (TI comfort OR AB comfort) OR (TI discomfort OR AB discomfort) OR (TI pain OR AB pain) OR (TI satisfaction OR AB satisfaction) OR (TI dissatisfaction OR AB dissatisfaction) OR (TI privacy OR AB privacy) OR (TI private OR AB private) OR (TI safe OR AB safe) OR (TI safety OR AB safety) OR (TI secure OR AB secure) OR (TI security OR AB security) OR (TI violent OR AB violent) OR (TI violence OR AB violence) OR (TI anxiety OR AB anxiety) OR (TI anxious OR AB anxious) OR (TI stress OR AB stress) OR (TI strain OR AB strain) OR (TI "self harm" OR AB "self harm") OR (TI "self injury" OR AB "self injury") OR (TI suicide OR AB suicide) OR (TI suicidal OR AB suicidal) OR (TI sedation OR AB sedation) OR (TI sedate OR AB sedate) OR (TI sedative OR AB sedative) OR (TI restrain OR AB restrain) OR (TI restraint OR AB restraint) OR (TI ligature OR AB ligature) OR (TI risk OR AB risk) OR (TI resilience OR AB resilience) OR (TI "length of stay" OR AB "length of stay") OR (TI "leaving without being seen" OR AB "leaving without being seen") OR (TI lwbs OR AB lwbs) OR (TI elope OR AB elope) OR (TI elopement OR AB elopement) OR (TI injure OR AB injure) OR (TI injury OR AB injury) OR (TI "family engagement" OR AB "family engagement") OR (TI transfer OR AB transfer) OR (TI wellbeing OR AB wellbeing) OR (TI "outpatient follow up" OR AB "outpatient follow up") OR (TI "ambulatory follow up" OR AB "ambulatory follow up"))
B) Built Environment	Setting	((MH "emergency service, hospital") OR (MH "emergency services, psychiatric") OR (TI "emergency department" OR AB "emergency department") OR (TI "emergency room" OR AB "emergency room") OR (TI "emergency service" OR AB "emergency service") OR (TI "emergency ward" OR AB "emergency ward") OR (TI "emergency clinic" OR AB "emergency clinic") OR (TI "emergency unit" OR AB "emergency unit") OR (TI "accident and emergency" OR AB "accident and emergency"))
	Population	**AND** (((MH "mental health") OR (MH "behavioral medicine") OR (MH "mental disorders") OR (TI "mental health" OR AB "mental health") OR (TI "behavioral health" OR AB "behavioral health") OR (TI psychiatry OR AB psychiatry) OR (TI psychology OR AB psychology) OR (TI psychiatric OR AB psychiatric) OR (TI autism OR AB autism) OR (TI "autism spectrum" OR AB "autism spectrum") OR (TI "cognitive impairment" OR AB "cognitive impairment") OR (TI "intellectual impairment" OR AB "intellectual impairment") OR (TI "cognitively impaired" OR AB "cognitively impaired") OR (TI "intellectually impaired" OR AB "intellectually impaired") OR (TI depression OR AB depression) OR (TI

		anxiety OR AB anxiety) OR (TI "attention deficit" OR AB "attention deficit") OR (TI hyperactivity OR AB hyperactivity) OR (TI adhd OR AB adhd) OR (TI "post traumatic stress" OR AB "post traumatic stress") OR (TI ptsd OR AB ptsd) OR (TI "obsessive compulsive" OR AB "obsessive compulsive") OR (TI tourette OR AB tourette) OR (TI "oppositional defiant" OR AB "oppositional defiant") OR (TI mania OR AB mania) OR (TI manic OR AB manic) OR (TI depressive OR AB depressive) OR (TI suicidal OR AB suicidal) OR (TI schizophrenia OR AB schizophrenia) OR (TI "schizophrenic" OR AB "schizophrenic") OR (TI "eating disorder" OR AB "eating disorder") OR (TI "anorexia" OR AB "anorexia") OR (TI "bulimia" OR AB "bulimia") OR (MH "substance-related disorders") OR (TI addiction OR AB addiction) OR (TI addicted OR AB addicted) OR (TI overdose OR AB overdose) OR (TI "substance abuse" OR AB "substance abuse") OR (TI "drug abuse" OR AB "drug abuse") OR (TI "alcohol abuse" OR AB "alcohol abuse") OR (TI intoxicated OR AB intoxicated) OR (TI intoxication OR AB intoxication)) **AND** ((MH "emergency nursing") OR (MH "emergency medicine") OR (MH "psychology") OR (MH "psychiatry") OR (MH "patients") OR (MH "family") OR (MH "parents") OR (MH "caregivers") OR (TI patient OR AB patient) OR (TI family OR AB family) OR (TI parent OR AB parent) OR (TI caregiver OR AB caregiver) OR (TI doctor OR AB doctor) OR (TI physician OR AB physician) OR (TI nurse OR AB nurse) OR (TI clinician OR AB clinician) OR (TI "healthcare worker" OR AB "healthcare worker") OR (TI psychiatrist OR AB psychiatrist) OR (TI psychologist OR AB psychologist) OR (TI "social worker" OR AB "social worker") OR (TI counselor OR AB counselor) OR (TI therapist OR AB therapist) OR (TI "care team" OR AB "care team") OR (TI security OR AB security) OR (TI ems OR AB ems)))
	Intervention	**AND** ((MH "built environment") OR (MH "hospital design and construction") OR (TI "built environment" OR AB "built environment") OR (TI "physical environment" OR AB "physical environment") OR (TI architecture OR AB architecture) OR (TI "interior design" OR AB "interior design") OR (TI lighting OR AB lighting) OR (TI daylight OR AB daylight) OR (TI window OR AB window) OR (TI "crisis stabilization" OR AB "crisis stabilization") OR (TI layout OR AB layout) OR (TI visibility OR AB visibility) OR (TI furniture OR AB furniture) OR (TI decoration OR AB decoration) OR (TI decor OR AB decor) OR (TI art OR AB art) OR (TI "positive distraction" OR AB "positive distraction") OR (TI noise OR AB noise) OR (TI ergonomics OR AB ergonomics) OR (TI ergonomic OR AB ergonomic) OR (TI odor OR AB odor) OR (TI smell OR AB smell) OR (TI "anti ligature" OR AB "anti ligature") OR (TI "ligature resistant" OR AB "ligature resistant") OR (TI "sensory room" OR AB "sensory room") OR (TI "snoezelen" OR AB "snoezelen"))
	Outcome	**AND** ((MH "defense mechanisms") OR (MH "emotions") OR (MH "behavioral symptoms") OR (MH "length of stay") OR (MH "patient participation") OR (MH "family relationships, professional") OR (TI aggression OR AB aggression) OR (TI aggressive OR AB aggressive) OR (TI agitation OR AB agitation) OR (TI agitated OR AB agitated) OR (TI confusion OR AB confusion) OR (TI distress OR AB distress) OR (TI anger OR AB anger) OR (TI angry OR AB angry) OR (TI comfort OR AB comfort) OR (TI discomfort OR AB discomfort) OR (TI pain OR AB pain) OR (TI satisfaction OR AB satisfaction) OR (TI dissatisfaction OR AB dissatisfaction) OR (TI privacy OR AB privacy) OR (TI private OR AB private) OR (TI safe OR AB safe) OR (TI safety OR AB safety) OR (TI secure OR AB secure) OR (TI security OR AB security) OR (TI violent OR AB violent) OR (TI violence OR AB violence) OR (TI anxiety OR AB anxiety) OR (TI anxious OR AB anxious) OR (TI stress OR AB stress) OR (TI strain OR AB strain) OR (TI "self harm" OR AB "self harm") OR (TI "self injury" OR AB "self injury") OR (TI suicide OR AB suicide) OR (TI suicidal OR AB suicidal) OR (TI sedation OR AB sedation) OR (TI sedate OR AB sedate) OR (TI sedative OR AB sedative) OR (TI restrain OR AB restrain) OR (TI restraint OR AB restraint) OR

		(TI ligature OR AB ligature) OR (TI risk OR AB risk) OR (TI resilience OR AB resilience) OR (TI "length of stay" OR AB "length of stay") OR (TI "leaving without being seen" OR AB "leaving without being seen") OR (TI lwbs OR AB lwbs) OR (TI elope OR AB elope) OR (TI elopement OR AB elopement) OR (TI injure OR AB injure) OR (TI injury OR AB injury) OR (TI "family engagement" OR AB "family engagement") OR (TI transfer OR AB transfer) OR (TI wellbeing OR AB wellbeing) OR (TI "outpatient follow up" OR AB "outpatient follow up") OR (TI "ambulatory follow up" OR AB "ambulatory follow up"))
C) Tools & Technology	Setting	((MH "emergency service, hospital") OR (MH "emergency services, psychiatric") OR (TI "emergency department" OR AB "emergency department") OR (TI "emergency room" OR AB "emergency room") OR (TI "emergency service" OR AB "emergency service") OR (TI "emergency ward" OR AB "emergency ward") OR (TI "emergency clinic" OR AB "emergency clinic") OR (TI "emergency unit" OR AB "emergency unit") OR (TI "accident and emergency" OR AB "accident and emergency"))
	Population	**AND** (((MH "mental health") OR (MH "behavioral medicine") OR (MH "mental disorders") OR (TI "mental health" OR AB "mental health") OR (TI "behavioral health" OR AB "behavioral health") OR (TI psychiatry OR AB psychiatry) OR (TI psychology OR AB psychology) OR (TI psychiatric OR AB psychiatric) OR (TI autism OR AB autism) OR (TI "autism spectrum" OR AB "autism spectrum") OR (TI "cognitive impairment" OR AB "cognitive impairment") OR (TI "intellectual impairment" OR AB "intellectual impairment") OR (TI "cognitively impaired" OR AB "cognitively impaired") OR (TI "intellectually impaired" OR AB "intellectually impaired") OR (TI depression OR AB depression) OR (TI anxiety OR AB anxiety) OR (TI "attention deficit" OR AB "attention deficit") OR (TI hyperactivity OR AB hyperactivity) OR (TI adhd OR AB adhd) OR (TI "post traumatic stress" OR AB "post traumatic stress") OR (TI ptsd OR AB ptsd) OR (TI "obsessive compulsive" OR AB "obsessive compulsive") OR (TI tourette OR AB tourette) OR (TI "oppositional defiant" OR AB "oppositional defiant") OR (TI mania OR AB mania) OR (TI manic OR AB manic) OR (TI depressive OR AB depressive) OR (TI suicidal OR AB suicidal) OR (TI schizophrenia OR AB schizophrenia) OR (TI "schizophrenic" OR AB "schizophrenic") OR (TI "eating disorder" OR AB "eating disorder") OR (TI "anorexia" OR AB "anorexia") OR (TI "bulimia" OR AB "bulimia") OR (MH "substance-related disorders") OR (TI addiction OR AB addiction) OR (TI addicted OR AB addicted) OR (TI overdose OR AB overdose) OR (TI "substance abuse" OR AB "substance abuse") OR (TI "drug abuse" OR AB "drug abuse") OR (TI "alcohol abuse" OR AB "alcohol abuse") OR (TI intoxicated OR AB intoxicated) OR (TI intoxication OR AB intoxication)) **AND** ((MH "emergency nursing") OR (MH "emergency medicine") OR (MH "psychology") OR (MH "psychiatry") OR (MH "patients") OR (MH "family") OR (MH "parents") OR (MH "caregivers") OR (TI patient OR AB patient) OR (TI family OR AB family) OR (TI parent OR AB parent) OR (TI caregiver OR AB caregiver) OR (TI doctor OR AB doctor) OR (TI physician OR AB physician) OR (TI nurse OR AB nurse) OR (TI clinician OR AB clinician) OR (TI "healthcare worker" OR AB "healthcare worker") OR (TI psychiatrist OR AB psychiatrist) OR (TI psychologist OR AB psychologist) OR (TI "social worker" OR AB "social worker") OR (TI counselor OR AB counselor) OR (TI therapist OR AB therapist) OR (TI "care team" OR AB "care team") OR (TI security OR AB security) OR (TI ems OR AB ems)))
	Intervention	((MH technology) OR (MH telemedicine) OR (TI email OR AB email) OR (TI "electronic mail" OR AB "electronic mail") OR (TI internet OR AB internet) OR (TI computer OR AB computer) OR (TI "medical informatics" OR AB "medical informatics") OR (TI software OR AB software) OR (TI telephone OR AB telephone) OR (TI technology OR AB technology) OR (TI kiosk OR AB kiosk) OR (TI "mobile app" OR AB "mobile app") OR (TI "mobile application" OR AB "mobile application") OR (TI wireless OR AB wireless) OR (TI mobile OR AB

		mobile) OR (TI “text message” OR AB “text message”) OR (TI message OR AB message) OR (TI messaging OR AB messaging) OR (TI sms OR AB sms) OR (TI “short messaging service” OR AB “short messaging service”) OR (TI mhealth OR AB mhealth) OR (TI ehealth OR AB ehealth) OR (TI handheld OR AB handheld) OR (TI laptop OR AB laptop) OR (TI palmtop OR AB palmtop) OR (TI tablet OR AB tablet) OR (TI smartphone OR AB smartphone) OR (TI “social network” OR AB “social network”) OR (TI “electronic health record” OR AB “electronic health record”) OR (TI “electronic medical record” OR AB “electronic medical record”) OR (TI ehr OR AB ehr) OR (TI emr OR AB emr) OR (TI telehealth OR AB telehealth) OR (TI telepsychiatry OR AB telepsychiatry) OR (TI telepsychology OR AB telepsychology) OR (TI simulation OR AB simulation))
	Outcome	**AND** ((MH "defense mechanisms") OR (MH “emotions”) OR (MH "behavioral symptoms") OR (MH "length of stay") OR (MH “patient participation”) OR (MH “family relationships, professional”) OR (TI aggression OR AB aggression) OR (TI aggressive OR AB aggressive) OR (TI agitation OR AB agitation) OR (TI agitated OR AB agitated) OR (TI confusion OR AB confusion) OR (TI distress OR AB distress) OR (TI anger OR AB anger) OR (TI angry OR AB angry) OR (TI comfort OR AB comfort) OR (TI discomfort OR AB discomfort) OR (TI pain OR AB pain) OR (TI satisfaction OR AB satisfaction) OR (TI dissatisfaction OR AB dissatisfaction) OR (TI privacy OR AB privacy) OR (TI private OR AB private) OR (TI safe OR AB safe) OR (TI safety OR AB safety) OR (TI secure OR AB secure) OR (TI security OR AB security) OR (TI violent OR AB violent) OR (TI violence OR AB violence) OR (TI anxiety OR AB anxiety) OR (TI anxious OR AB anxious) OR (TI stress OR AB stress) OR (TI strain OR AB strain) OR (TI "self harm" OR AB "self harm") OR (TI "self injury" OR AB "self injury") OR (TI suicide OR AB suicide) OR (TI suicidal OR AB suicidal) OR (TI sedation OR AB sedation) OR (TI sedate OR AB sedate) OR (TI sedative OR AB sedative) OR (TI restrain OR AB restrain) OR (TI restraint OR AB restraint) OR (TI ligature OR AB ligature) OR (TI risk OR AB risk) OR (TI resilience OR AB resilience) OR (TI "length of stay" OR AB "length of stay") OR (TI "leaving without being seen" OR AB "leaving without being seen") OR (TI lwbs OR AB lwbs) OR (TI elope OR AB elope) OR (TI elopement OR AB elopement) OR (TI injure OR AB injure) OR (TI injury OR AB injury) OR (TI "family engagement" OR AB "family engagement") OR (TI transfer OR AB transfer) OR (TI wellbeing OR AB wellbeing) OR (TI “outpatient follow up” OR AB “outpatient follow up”) OR (TI “ambulatory follow up” OR AB “ambulatory follow up”))
APA PsycInfo		
A) Tasks & Organizational Conditions	Setting	((SU "emergency services") OR (TI "emergency department" OR AB "emergency department") OR (TI "emergency room" OR AB "emergency room") OR (TI "emergency service" OR AB "emergency service") OR (TI "emergency ward" OR AB "emergency ward") OR (TI "emergency clinic" OR AB "emergency clinic") OR (TI "emergency unit" OR AB "emergency unit") OR (TI "accident and emergency" OR AB "accident and emergency"))
	Population	**AND** (((SU "mental health") OR (SU "behavioral medicine") OR (SU “mental disorders”) OR (TI "mental health" OR AB "mental health") OR (TI "behavioral health" OR AB "behavioral health") OR (TI psychiatry OR AB psychiatry) OR (TI psychology OR AB psychology) OR (TI psychiatric OR AB psychiatric) OR (TI autism OR AB autism) OR (TI "autism spectrum" OR AB "autism spectrum") OR (TI "cognitive impairment" OR AB "cognitive impairment") OR (TI "intellectual impairment" OR AB "intellectual impairment") OR (TI "cognitively impaired" OR AB "cognitively impaired") OR (TI "intellectually impaired" OR AB "intellectually impaired") OR (TI depression OR AB depression) OR (TI anxiety OR AB anxiety) OR (TI "attention deficit" OR AB "attention deficit") OR (TI hyperactivity OR AB hyperactivity) OR (TI adhd OR AB adhd) OR (TI "post traumatic stress" OR AB "post traumatic stress") OR (TI ptsd OR AB ptsd)

		OR (TI "obsessive compulsive" OR AB "obsessive compulsive") OR (TI tourette OR AB tourette) OR (TI "oppositional defiant" OR AB "oppositional defiant") OR (TI mania OR AB mania) OR (TI manic OR AB manic) OR (TI depressive OR AB depressive) OR (TI suicidal OR AB suicidal) OR (TI schizophrenia OR AB schizophrenia) OR (TI "schizophrenic" OR AB "schizophrenic") OR (TI "eating disorder" OR AB "eating disorder") OR (TI "anorexia" OR AB "anorexia") OR (TI "bulimia" OR AB "bulimia") OR (SU "drug abuse") OR (TI addiction OR AB addiction) OR (TI addicted OR AB addicted) OR (TI overdose OR AB overdose) OR (TI "substance abuse" OR AB "substance abuse") OR (TI "drug abuse" OR AB "drug abuse") OR (TI "alcohol abuse" OR AB "alcohol abuse") OR (TI intoxicated OR AB intoxicated) OR (TI intoxication OR AB intoxication)) **AND** ((SU "emergency personnel") OR (SU "emergency medicine") OR (SU psychology) OR (SU psychiatry) OR (SU "mental health personnel") OR (SU patients) OR (SU "psychiatric patients") OR (SU family) OR (SU parents) OR (SU caregivers) OR (TI patient OR AB patient) OR (TI family OR AB family) OR (TI parent OR AB parent) OR (TI caregiver OR AB caregiver) OR (TI doctor OR AB doctor) OR (TI physician OR AB physician) OR (TI nurse OR AB nurse) OR (TI clinician OR AB clinician) OR (TI "healthcare worker" OR AB "healthcare worker") OR (TI psychiatrist OR AB psychiatrist) OR (TI psychologist OR AB psychologist) OR (TI "social worker" OR AB "social worker") OR (TI counselor OR AB counselor) OR (TI therapist OR AB therapist) OR (TI "care team" OR AB "care team") OR (TI security OR AB security) OR (TI ems OR AB ems)))
	Intervention	**AND** ((SU "continuing education") OR (SU "medical education") OR (SU "nursing education") OR (SU "decision making") OR (TI task OR AB task) OR (TI process OR AB process) OR (TI workflow OR AB workflow) OR (TI triage OR AB triage) OR (TI "fast track" OR AB "fast track") OR (TI lean OR AB lean) OR (TI treatment OR AB treatment) OR (TI assess OR AB assess) OR (TI assessment OR AB assessment) OR (TI screening OR AB screening) OR (TI "medical screening" OR AB "medical screening") OR (TI "psychiatric screening" OR AB "psychiatric screening") OR (TI evaluation OR AB evaluation) OR (TI evaluate OR AB evaluate) OR (TI training OR AB training) OR (TI "professional development" OR AB "professional development") OR (TI mentor OR AB mentor) OR (TI mentorship OR AB mentorship) OR (TI learning OR AB learning) OR (TI education OR AB education) OR (TI culture OR AB culture) OR (TI management OR AB management) OR (TI manager OR AB manager) OR (TI change OR AB change) OR (TI leader OR AB leader) OR (TI leadership OR AB leadership) OR (TI team OR AB team) OR (TI teamwork OR AB teamwork) OR (TI policy OR AB policy) OR (TI policies OR AB policies) OR (TI staffing OR AB staffing) OR (TI scheduling OR AB scheduling) OR (TI schedule OR AB schedule))
	Outcome	**AND** ((SU "defense mechanisms") OR (SU emotions) OR (SU "treatment duration") OR (SU "client participation") OR (TI aggression OR AB aggression) OR (TI aggressive OR AB aggressive) OR (TI agitation OR AB agitation) OR (TI agitated OR AB agitated) OR (TI confusion OR AB confusion) OR (TI distress OR AB distress) OR (TI anger OR AB anger) OR (TI angry OR AB angry) OR (TI comfort OR AB comfort) OR (TI discomfort OR AB discomfort) OR (TI pain OR AB pain) OR (TI satisfaction OR AB satisfaction) OR (TI dissatisfaction OR AB dissatisfaction) OR (TI privacy OR AB privacy) OR (TI private OR AB private) OR (TI safe OR AB safe) OR (TI safety OR AB safety) OR (TI secure OR AB secure) OR (TI security OR AB security) OR (TI violent OR AB violent) OR (TI violence OR AB violence) OR (TI anxiety OR AB anxiety) OR (TI anxious OR AB anxious) OR (TI stress OR AB stress) OR (TI strain OR AB strain) OR (TI "self harm" OR AB "self harm") OR (TI "self injury" OR AB "self injury") OR (TI suicide OR AB suicide) OR (TI suicidal OR AB suicidal) OR (TI sedation OR AB sedation) OR (TI sedate OR AB sedate)

		OR (TI sedative OR AB sedative) OR (TI restrain OR AB restrain) OR (TI restraint OR AB restraint) OR (TI ligature OR AB ligature) OR (TI risk OR AB risk) OR (TI resilience OR AB resilience) OR (TI "length of stay" OR AB "length of stay") OR (TI "leaving without being seen" OR AB "leaving without being seen") OR (TI lwbs OR AB lwbs) OR (TI elope OR AB elope) OR (TI elopement OR AB elopement) OR (TI injure OR AB injure) OR (TI injury OR AB injury) OR (TI "family engagement" OR AB "family engagement") OR (TI transfer OR AB transfer) OR (TI wellbeing OR AB wellbeing) OR (TI “outpatient follow up” OR AB “outpatient follow up”) OR (TI “ambulatory follow up” OR AB “ambulatory follow up”))
B) Built Environment	Setting	((SU "emergency services") OR (TI "emergency department" OR AB "emergency department") OR (TI "emergency room" OR AB "emergency room") OR (TI "emergency service" OR AB "emergency service") OR (TI "emergency ward" OR AB "emergency ward") OR (TI "emergency clinic" OR AB "emergency clinic") OR (TI "emergency unit" OR AB "emergency unit") OR (TI "accident and emergency" OR AB "accident and emergency"))
	Population	**AND** (((SU "mental health") OR (SU "behavioral medicine") OR (SU “mental disorders”) OR (TI "mental health" OR AB "mental health") OR (TI "behavioral health" OR AB "behavioral health") OR (TI psychiatry OR AB psychiatry) OR (TI psychology OR AB psychology) OR (TI psychiatric OR AB psychiatric) OR (TI autism OR AB autism) OR (TI "autism spectrum" OR AB "autism spectrum") OR (TI "cognitive impairment" OR AB "cognitive impairment") OR (TI "intellectual impairment" OR AB "intellectual impairment") OR (TI "cognitively impaired" OR AB "cognitively impaired") OR (TI "intellectually impaired" OR AB "intellectually impaired") OR (TI depression OR AB depression) OR (TI anxiety OR AB anxiety) OR (TI "attention deficit" OR AB "attention deficit") OR (TI hyperactivity OR AB hyperactivity) OR (TI adhd OR AB adhd) OR (TI "post traumatic stress" OR AB "post traumatic stress") OR (TI ptsd OR AB ptsd) OR (TI "obsessive compulsive" OR AB "obsessive compulsive") OR (TI tourette OR AB tourette) OR (TI "oppositional defiant" OR AB "oppositional defiant") OR (TI mania OR AB mania) OR (TI manic OR AB manic) OR (TI depressive OR AB depressive) OR (TI suicidal OR AB suicidal) OR (TI schizophrenia OR AB schizophrenia) OR (TI "schizophrenic" OR AB “schizophrenic”) OR (TI “eating disorder” OR AB “eating disorder”) OR (TI “anorexia” OR AB “anorexia”) OR (TI “bulimia” OR AB “bulimia”) OR (SU “drug abuse”) OR (TI addiction OR AB addiction) OR (TI addicted OR AB addicted) OR (TI overdose OR AB overdose) OR (TI “substance abuse” OR AB “substance abuse”) OR (TI “drug abuse” OR AB “drug abuse”) OR (TI “alcohol abuse” OR AB “alcohol abuse”) OR (TI intoxicated OR AB intoxicated) OR (TI intoxication OR AB intoxication)) **AND** ((SU "emergency personnel") OR (SU "emergency medicine") OR (SU psychology) OR (SU psychiatry) OR (SU “mental health personnel”) OR (SU patients) OR (SU “psychiatric patients”) OR (SU family) OR (SU parents) OR (SU caregivers) OR (TI patient OR AB patient) OR (TI family OR AB family) OR (TI parent OR AB parent) OR (TI caregiver OR AB caregiver) OR (TI doctor OR AB doctor) OR (TI physician OR AB physician) OR (TI nurse OR AB nurse) OR (TI clinician OR AB clinician) OR (TI "healthcare worker" OR AB "healthcare worker") OR (TI psychiatrist OR AB psychiatrist) OR (TI psychologist OR AB psychologist) OR (TI "social worker" OR AB "social worker") OR (TI counselor OR AB counselor) OR (TI therapist OR AB therapist) OR (TI "care team" OR AB "care team") OR (TI security OR AB security) OR (TI ems OR AB ems)))
	Intervention	**AND** ((SU “built environment”) OR (TI “built environment” OR AB “built environment”) OR (TI “physical environment” OR AB “physical environment”) OR (TI architecture OR AB architecture) OR (TI “interior design” OR AB “interior design”) OR (TI lighting OR AB lighting) OR (TI daylight OR AB daylight) OR (TI window OR AB window) OR (TI “crisis stabilization” OR AB

		“crisis stabilization”) OR (TI layout OR AB layout) OR (TI visibility OR AB visibility) OR (TI furniture OR AB furniture) OR (TI decoration OR AB decoration) OR (TI decor OR AB decor) OR (TI art OR AB art) OR (TI “positive distraction” OR AB “positive distraction”) OR (TI noise OR AB noise) OR (TI ergonomics OR AB ergonomics) OR (TI ergonomic OR AB ergonomic) OR (TI odor OR AB odor) OR (TI smell OR AB smell) OR (TI “anti ligature” OR AB “anti ligature”) OR (TI “ligature resistant” OR AB “ligature resistant”) OR (TI “sensory room” OR AB “sensory room”) OR (TI “snoezelen” OR AB “snoezelen”))
	Outcome	**AND** ((SU "defense mechanisms") OR (SU emotions) OR (SU "treatment duration") OR (SU “client participation”) OR (TI aggression OR AB aggression) OR (TI aggressive OR AB aggressive) OR (TI agitation OR AB agitation) OR (TI agitated OR AB agitated) OR (TI confusion OR AB confusion) OR (TI distress OR AB distress) OR (TI anger OR AB anger) OR (TI angry OR AB angry) OR (TI comfort OR AB comfort) OR (TI discomfort OR AB discomfort) OR (TI pain OR AB pain) OR (TI satisfaction OR AB satisfaction) OR (TI dissatisfaction OR AB dissatisfaction) OR (TI privacy OR AB privacy) OR (TI private OR AB private) OR (TI safe OR AB safe) OR (TI safety OR AB safety) OR (TI secure OR AB secure) OR (TI security OR AB security) OR (TI violent OR AB violent) OR (TI violence OR AB violence) OR (TI anxiety OR AB anxiety) OR (TI anxious OR AB anxious) OR (TI stress OR AB stress) OR (TI strain OR AB strain) OR (TI "self harm" OR AB "self harm") OR (TI "self injury" OR AB "self injury") OR (TI suicide OR AB suicide) OR (TI suicidal OR AB suicidal) OR (TI sedation OR AB sedation) OR (TI sedate OR AB sedate) OR (TI sedative OR AB sedative) OR (TI restrain OR AB restrain) OR (TI restraint OR AB restraint) OR (TI ligature OR AB ligature) OR (TI risk OR AB risk) OR (TI resilience OR AB resilience) OR (TI "length of stay" OR AB "length of stay") OR (TI "leaving without being seen" OR AB "leaving without being seen") OR (TI lwbs OR AB lwbs) OR (TI elope OR AB elope) OR (TI elopement OR AB elopement) OR (TI injure OR AB injure) OR (TI injury OR AB injury) OR (TI "family engagement" OR AB "family engagement") OR (TI transfer OR AB transfer) OR (TI wellbeing OR AB wellbeing) OR (TI “outpatient follow up” OR AB “outpatient follow up”) OR (TI “ambulatory follow up” OR AB “ambulatory follow up”))
C) Tools & Technology	Setting	((SU "emergency services") OR (TI "emergency department" OR AB "emergency department") OR (TI "emergency room" OR AB "emergency room") OR (TI "emergency service" OR AB "emergency service") OR (TI "emergency ward" OR AB "emergency ward") OR (TI "emergency clinic" OR AB "emergency clinic") OR (TI "emergency unit" OR AB "emergency unit") OR (TI "accident and emergency" OR AB "accident and emergency"))
	Population	**AND** (((SU "mental health") OR (SU "behavioral medicine") OR (SU “mental disorders”) OR (TI "mental health" OR AB "mental health") OR (TI "behavioral health" OR AB "behavioral health") OR (TI psychiatry OR AB psychiatry) OR (TI psychology OR AB psychology) OR (TI psychiatric OR AB psychiatric) OR (TI autism OR AB autism) OR (TI "autism spectrum" OR AB "autism spectrum") OR (TI "cognitive impairment" OR AB "cognitive impairment") OR (TI "intellectual impairment" OR AB "intellectual impairment") OR (TI "cognitively impaired" OR AB "cognitively impaired") OR (TI "intellectually impaired" OR AB "intellectually impaired") OR (TI depression OR AB depression) OR (TI anxiety OR AB anxiety) OR (TI "attention deficit" OR AB "attention deficit") OR (TI hyperactivity OR AB hyperactivity) OR (TI adhd OR AB adhd) OR (TI "post traumatic stress" OR AB "post traumatic stress") OR (TI ptsd OR AB ptsd) OR (TI "obsessive compulsive" OR AB "obsessive compulsive") OR (TI tourette OR AB tourette) OR (TI "oppositional defiant" OR AB "oppositional defiant") OR (TI mania OR AB mania) OR (TI manic OR AB manic) OR (TI depressive OR AB depressive) OR (TI suicidal OR AB suicidal) OR (TI schizophrenia OR

		AB schizophrenia) OR (TI "schizophrenic" OR AB "schizophrenic") OR (TI "eating disorder" OR AB "eating disorder") OR (TI "anorexia" OR AB "anorexia") OR (TI "bulimia" OR AB "bulimia") OR (SU "drug abuse") OR (TI addiction OR AB addiction) OR (TI addicted OR AB addicted) OR (TI overdose OR AB overdose) OR (TI "substance abuse" OR AB "substance abuse") OR (TI "drug abuse" OR AB "drug abuse") OR (TI "alcohol abuse" OR AB "alcohol abuse") OR (TI intoxicated OR AB intoxicated) OR (TI intoxication OR AB intoxication)) **AND** ((SU "emergency personnel") OR (SU "emergency medicine") OR (SU psychology) OR (SU psychiatry) OR (SU "mental health personnel") OR (SU patients) OR (SU "psychiatric patients") OR (SU family) OR (SU parents) OR (SU caregivers) OR (TI patient OR AB patient) OR (TI family OR AB family) OR (TI parent OR AB parent) OR (TI caregiver OR AB caregiver) OR (TI doctor OR AB doctor) OR (TI physician OR AB physician) OR (TI nurse OR AB nurse) OR (TI clinician OR AB clinician) OR (TI "healthcare worker" OR AB "healthcare worker") OR (TI psychiatrist OR AB psychiatrist) OR (TI psychologist OR AB psychologist) OR (TI "social worker" OR AB "social worker") OR (TI counselor OR AB counselor) OR (TI therapist OR AB therapist) OR (TI "care team" OR AB "care team") OR (TI security OR AB security) OR (TI ems OR AB ems)))
	Intervention	**AND** ((SU technology) OR (SU telemedicine) OR (TI email OR AB email) OR (TI "electronic mail" OR AB "electronic mail") OR (TI internet OR AB internet) OR (TI computer OR AB computer) OR (TI "medical informatics" OR AB "medical informatics") OR (TI software OR AB software) OR (TI telephone OR AB telephone) OR (TI technology OR AB technology) OR (TI kiosk OR AB kiosk) OR (TI "mobile app" OR AB "mobile app") OR (TI "mobile application" OR AB "mobile application") OR (TI wireless OR AB wireless) OR (TI mobile OR AB mobile) OR (TI "text message" OR AB "text message") OR (TI message OR AB message) OR (TI messaging OR AB messaging) OR (TI sms OR AB sms) OR (TI "short messaging service" OR AB "short messaging service") OR (TI mhealth OR AB mhealth) OR (TI ehealth OR AB ehealth) OR (TI handheld OR AB handheld) OR (TI laptop OR AB laptop) OR (TI palmtop OR AB palmtop) OR (TI tablet OR AB tablet) OR (TI smartphone OR AB smartphone) OR (TI "social network" OR AB "social network") OR (TI "electronic health record" OR AB "electronic health record") OR (TI "electronic medical record" OR AB "electronic medical record") OR (TI ehr OR AB ehr) OR (TI emr OR AB emr) OR (TI telehealth OR AB telehealth) OR (TI telepsychiatry OR AB telepsychiatry) OR (TI telepsychology OR AB telepsychology) OR (TI simulation OR AB simulation))
	Outcome	**AND** ((SU "defense mechanisms") OR (SU emotions) OR (SU "treatment duration") OR (SU "client participation") OR (TI aggression OR AB aggression) OR (TI aggressive OR AB aggressive) OR (TI agitation OR AB agitation) OR (TI agitated OR AB agitated) OR (TI confusion OR AB confusion) OR (TI distress OR AB distress) OR (TI anger OR AB anger) OR (TI angry OR AB angry) OR (TI comfort OR AB comfort) OR (TI discomfort OR AB discomfort) OR (TI pain OR AB pain) OR (TI satisfaction OR AB satisfaction) OR (TI dissatisfaction OR AB dissatisfaction) OR (TI privacy OR AB privacy) OR (TI private OR AB private) OR (TI safe OR AB safe) OR (TI safety OR AB safety) OR (TI secure OR AB secure) OR (TI security OR AB security) OR (TI violent OR AB violent) OR (TI violence OR AB violence) OR (TI anxiety OR AB anxiety) OR (TI anxious OR AB anxious) OR (TI stress OR AB stress) OR (TI strain OR AB strain) OR (TI "self harm" OR AB "self harm") OR (TI "self injury" OR AB "self injury") OR (TI suicide OR AB suicide) OR (TI suicidal OR AB suicidal) OR (TI sedation OR AB sedation) OR (TI sedate OR AB sedate) OR (TI sedative OR AB sedative) OR (TI restrain OR AB restrain) OR (TI restraint OR AB restraint) OR (TI ligature OR AB ligature) OR (TI risk OR AB risk) OR (TI resilience OR AB resilience) OR (TI "length of stay" OR AB

		"length of stay") OR (TI "leaving without being seen" OR AB "leaving without being seen") OR (TI lwbs OR AB lwbs) OR (TI elope OR AB elope) OR (TI elopement OR AB elopement) OR (TI injure OR AB injure) OR (TI injury OR AB injury) OR (TI "family engagement" OR AB "family engagement") OR (TI transfer OR AB transfer) OR (TI wellbeing OR AB wellbeing) OR (TI "outpatient follow up" OR AB "outpatient follow up") OR (TI "ambulatory follow up" OR AB "ambulatory follow up"))
Web of Science Core Collection		
A) Tasks & Organizational Conditions	Setting	(TI=(emergency service) OR TI=(psychiatric emergency service) OR TI=(emergency department) OR TI=(emergency room) OR TI=(emergency ward) OR TI=(emergency clinic) OR TI=(emergency unit) OR TI=(accident and emergency) **OR** AB=(emergency service) OR AB=(psychiatric emergency service) OR AB=(emergency department) OR AB=(emergency room) OR AB=(emergency ward) OR AB=(emergency clinic) OR AB=(emergency unit) OR AB=(accident and emergency))
	Population	**AND** ((TI=(mental health) OR TI=(behavioral and mental disorders) OR TI=(behavioral health) OR TI=(psychiatry) OR TI=(psychology) OR TI=(psychiatric) OR TI=(autism) OR TI=(autism spectrum) OR TI=(cognitive impairment) OR TI=(intellectual impairment) OR TI=(cognitively impaired) OR TI=(intellectually impaired) OR TI=(depression) OR TI=(anxiety) OR TI=(attention deficit) OR TI=(hyperactivity) OR TI=(adhd) OR TI=(post traumatic stress) OR TI=(ptsd) OR TI=(obsessive compulsive) OR TI=(Tourette) OR TI=(mania) OR TI=(manic) OR TI=(depressive) OR TI=(suicidal) OR TI=(schizophrenia) OR TI=(schizophrenic) OR TI=(eating disorder) OR TI=(anorexia) OR TI=(bulimia) OR TI=(addiction) OR TI=(addicted) OR TI=(overdose) OR TI=(substance abuse) OR TI=(drug abuse) OR TI=(alcohol abuse) OR TI=(intoxicated) OR TI=(intoxication) **OR** AB=(mental health) OR AB=(behavioral and mental disorders) OR AB=(behavioral health) OR AB=(psychiatry) OR AB=(psychology) OR AB=(psychiatric) OR AB=(autism) OR AB=(autism spectrum) OR AB=(cognitive impairment) OR AB=(intellectual impairment) OR AB=(cognitively impaired) OR AB=(intellectually impaired) OR AB=(depression) OR AB=(anxiety) OR AB=(attention deficit) OR AB=(hyperactivity) OR AB=(adhd) OR AB=(post traumatic stress) OR AB=(ptsd) OR AB=(obsessive compulsive) OR AB=(Tourette) OR AB=(mania) OR AB=(manic) OR AB=(depressive) OR AB=(suicidal) OR AB=(schizophrenia) OR AB=(schizophrenic) OR AB=(eating disorder) OR AB=(anorexia) OR AB=(bulimia) OR AB=(addiction) OR AB=(addicted) OR AB=(overdose) OR AB=(substance abuse) OR AB=(drug abuse) OR AB=(alcohol abuse) OR AB=(intoxicated) OR AB=(intoxication)) **AND** (TI=(emergency nursing) OR TI=(emergency medicine) OR TI=(mental health personnel) OR TI=(patient) OR TI=(psychiatric patient) OR TI=(family) OR TI=(parent) OR TI =(caregiver) OR TI=(doctor) OR TI=(physician) OR TI=(nurse) OR TI=(clinician) OR TI=(healthcare worker) OR TI=(psychiatrist) OR TI=(psychologist) OR TI=(social worker) OR TI=(counselor) OR TI=(therapist) OR TI=(care team) OR TI=(security) OR TI=(ems) **OR** AB=(emergency nursing) OR AB=(emergency medicine) OR AB=(mental health personnel) OR AB=(patient) OR AB=(family) OR AB=(parent) OR AB=(caregiver) OR AB=(psychiatric patient) OR AB=(doctor) OR AB=(physician) OR AB=(nurse) OR AB=(clinician) OR AB=(healthcare worker) OR AB=(psychiatrist) OR AB=(psychologist) OR AB=(social worker) OR AB=(counselor) OR AB=(therapist) OR AB=(care team) OR AB=(security) OR AB=(ems)))
	Intervention	**AND** (TI=(continuing education) OR TI=(education) OR TI=(workflow) OR TI=(decision making) OR TI=(task) OR TI=(process) OR TI=(triage) OR TI=(fast track) OR TI=(lean) OR TI=(treatment) OR TI=(assess) OR TI=(assessment) OR TI=(screening) OR TI=(medical screening) OR

		TI=(psychiatric screening) OR TI=(evaluation) OR TI=(evaluate) OR TI=(training) OR TI=(professional development) OR TI=(mentor) OR TI=(mentorship) OR TI=(learning) OR TI=(culture) OR TI=(management) OR TI=(manager) OR TI=(change) OR TI=(leader) OR TI=(leadership) OR TI=(team) OR TI=(teamwork) OR TI=(policy) OR TI=(policies) OR TI=(staffing) OR TI=(scheduling) OR TI=(schedule) **OR** AB=(continuing education) OR AB=(education) OR AB=(workflow) OR AB=(decision making) OR AB=(task) OR AB=(process) OR AB=(triage) OR AB=(fast track) OR AB=(lean) OR AB=(treatment) OR AB=(assess) OR AB=(assessment) OR AB=(screening) OR AB=(medical screening) OR AB=(psychiatric screening) OR AB=(evaluation) OR AB=(evaluate) OR AB=(training) OR AB=(professional development) OR AB=(mentor) OR AB=(mentorship) OR AB=(learning) OR AB=(culture) OR AB=(management) OR AB=(manager) OR AB=(change) OR AB=(leader) OR AB=(leadership) OR AB=(team) OR AB=(teamwork) OR AB=(policy) OR AB=(policies) OR AB=(staffing) OR AB=(scheduling) OR AB=(schedule))
	Outcome	**AND** (TI=(defense mechanisms) OR TI=(emotions) OR TI=(behavioral symptoms) OR TI=(length of stay) OR TI=(patient relations) OR TI=(family relations) OR TI=(aggression) OR TI=(aggressive) OR TI=(aggressive) OR TI=(agitated) OR TI=(confusion) OR TI=(distress) OR TI=(anger) OR TI=(angry) OR TI=(comfort) OR TI=(discomfort) OR TI=(pain) OR TI=(satisfaction) OR TI=(dissatisfaction) OR TI=(privacy) OR TI=(private) OR TI=(safe) OR TI=(safety) OR TI=(secure) OR TI=(security) OR TI=(violent) OR TI=(violence) OR TI=(anxiety) OR TI=(anxious) OR TI=(stress) OR TI=(strain) OR TI=(self harm) OR TI=(self injury) OR TI=(suicide) OR TI=(suicidal) OR TI=(sedation) OR TI=(sedate) OR TI=(sedative) OR TI=(restrain) OR TI=(restraint) OR TI=(ligature) OR TI=(risk) OR TI=(resilience) OR TI=(length of stay) OR TI=(leaving without being seen) OR TI=(lwbs) OR TI=(elope) OR TI=(elopement) OR TI=(injure) OR TI=(injury) OR TI=(family engagement) OR TI=(transfer) OR TI=(wellbeing) OR TI=(outpatient follow up) OR TI=(ambulatory follow up) **OR** AB=(defense mechanisms) OR AB=(emotions) OR AB=(behavioral symptoms) OR AB=(length of stay) OR AB=(patient relations) OR AB=(family relations) OR AB=(aggression) OR AB=(aggressive) OR AB=(aggressive) OR AB=(agitated) OR AB=(confusion) OR AB=(distress) OR AB=(anger) OR AB=(angry) OR AB=(comfort) OR AB=(discomfort) OR AB=(pain) OR AB=(satisfaction) OR AB=(dissatisfaction) OR AB=(privacy) OR AB=(private) OR AB=(safe) OR AB=(safety) OR AB=(secure) OR AB=(security) OR AB=(violent) OR AB=(violence) OR AB=(anxiety) OR AB=(anxious) OR AB=(stress) OR AB=(strain) OR AB=(self harm) OR AB=(self injury) OR AB=(suicide) OR AB=(suicidal) OR AB=(sedation) OR AB=(sedate) OR AB=(sedative) OR AB=(restrain) OR AB=(restraint) OR AB=(ligature) OR AB=(risk) OR AB=(resilience) OR AB=(length of stay) OR AB=(leaving without being seen) OR AB=(lwbs) OR AB=(elope) OR AB=(elopement) OR AB=(injure) OR AB=(injury) OR AB=(family engagement) OR AB=(transfer) OR AB=(wellbeing) OR AB=(outpatient follow up) OR AB=(ambulatory follow up))
B) Built Environment	Setting	(TI=(emergency service) OR TI=(psychiatric emergency service) OR TI=(emergency department) OR TI=(emergency room) OR TI=(emergency ward) OR TI=(emergency clinic) OR TI=(emergency unit) OR TI=(accident and emergency) **OR** AB=(emergency service) OR AB=(psychiatric emergency service) OR AB=(emergency department) OR AB=(emergency room) OR AB=(emergency ward) OR AB=(emergency clinic) OR AB=(emergency unit) OR AB=(accident and emergency))
	Population	**AND** ((TI=(mental health) OR TI=(behavioral and mental disorders) OR TI=(behavioral health) OR TI=(psychiatry) OR TI=(psychology) OR TI=(psychiatric) OR TI=(autism) OR TI=(autism spectrum) OR TI=(cognitive

		impairment) OR TI=(intellectual impairment) OR TI=(cognitively impaired) OR TI=(intellectually impaired) OR TI=(depression) OR TI=(anxiety) OR TI=(attention deficit) OR TI=(hyperactivity) OR TI=(adhd) OR TI=(post traumatic stress) OR TI=(ptsd) OR TI=(obsessive compulsive) OR TI=(Tourette) OR TI=(mania) OR TI=(manic) OR TI=(depressive) OR TI=(suicidal) OR TI=(schizophrenia) OR TI=(schizophrenic) OR TI=(eating disorder) OR TI=(anorexia) OR TI=(bulimia) OR TI=(addiction) OR TI=(addicted) OR TI=(overdose) OR TI=(substance abuse) OR TI=(drug abuse) OR TI=(alcohol abuse) OR TI=(intoxicated) OR TI=(intoxication) **OR** AB=(mental health) OR AB=(behavioral and mental disorders) OR AB=(behavioral health) OR AB=(psychiatry) OR AB=(psychology) OR AB=(psychiatric) OR AB=(autism) OR AB=(autism spectrum) OR AB=(cognitive impairment) OR AB=(intellectual impairment) OR AB=(cognitively impaired) OR AB=(intellectually impaired) OR AB=(depression) OR AB=(anxiety) OR AB=(attention deficit) OR AB=(hyperactivity) OR AB=(adhd) OR AB=(post traumatic stress) OR AB=(ptsd) OR AB=(obsessive compulsive) OR AB=(Tourette) OR AB=(mania) OR AB=(manic) OR AB=(depressive) OR AB=(suicidal) OR AB=(schizophrenia) OR AB=(schizophrenic) OR AB=(eating disorder) OR AB=(anorexia) OR AB=(bulimia) OR AB=(addiction) OR AB=(addicted) OR AB=(overdose) OR AB=(substance abuse) OR AB=(drug abuse) OR AB=(alcohol abuse) OR AB=(intoxicated) OR AB=(intoxication)) **AND** (TI=(emergency nursing) OR TI=(emergency medicine) OR TI=(mental health personnel) OR TI=(patient) OR TI=(psychiatric patient) OR TI=(family) OR TI=(parent) OR TI =(caregiver) OR TI=(doctor) OR TI=(physician) OR TI=(nurse) OR TI=(clinician) OR TI=(healthcare worker) OR TI=(psychiatrist) OR TI=(psychologist) OR TI=(social worker) OR TI=(counselor) OR TI=(therapist) OR TI=(care team) OR TI=(security) OR TI=(ems) **OR** AB=(emergency nursing) OR AB=(emergency medicine) OR AB=(mental health personnel) OR AB=(patient) OR AB=(family) OR AB=(parent) OR AB=(caregiver) OR AB=(psychiatric patient) OR AB=(doctor) OR AB=(physician) OR AB=(nurse) OR AB=(clinician) OR AB=(healthcare worker) OR AB=(psychiatrist) OR AB=(psychologist) OR AB=(social worker) OR AB=(counselor) OR AB=(therapist) OR AB=(care team) OR AB=(security) OR AB=(ems)))
	Intervention	**AND** (TI=(built environment) OR TI=(hospital design) OR TI=(construction) OR TI=(built environment) OR TI=(physical environment) OR TI=(architecture) OR TI=(interior design) OR TI=(lighting) OR TI=(daylight) OR TI=(window) OR TI=(crisis stabilization) OR TI=(layout) OR TI=(visibility) OR TI=(furniture) OR TI=(decoration) OR TI=(décor) OR TI=(art) OR TI=(positive distraction) OR TI=(noise) OR TI=(ergonomics) OR TI=(ergonomic) OR TI=(odor) OR TI=(smell) OR TI=(anti ligature) OR TI=(ligature resistant) OR TI=(sensory room) OR TI=(Snoezelen) **OR** AB=(built environment) OR AB=(hospital design) OR AB=(construction) OR AB=(built environment) OR AB=(physical environment) OR AB=(architecture) OR AB=(interior design) OR AB=(lighting) OR TI=(daylight) OR AB=(window) OR AB=(crisis stabilization) OR AB=(layout) OR AB=(visibility) OR AB=(furniture) OR AB=(decoration) OR AB=(décor) OR AB=(art) OR AB=(positive distraction) OR AB=(noise) OR AB=(ergonomics) OR AB=(ergonomic) OR AB=(odor) OR AB=(smell) OR AB=(anti ligature) OR AB=(ligature resistant) OR AB=(sensory room) OR AB=(Snoezelen))
	Outcome	**AND** (TI=(defense mechanisms) OR TI=(emotions) OR TI=(behavioral symptoms) OR TI=(length of stay) OR TI=(patient relations) OR TI=(family relations) OR TI=(aggression) OR TI=(aggressive) OR TI=(aggressive) OR TI=(agitated) OR TI=(confusion) OR TI=(distress) OR TI=(anger) OR TI=(angry) OR TI=(comfort) OR TI=(discomfort) OR TI=(pain) OR TI=(satisfaction) OR TI=(dissatisfaction) OR TI=(privacy) OR TI=(private) OR

		TI=(safe) OR TI=(safety) OR TI=(secure) OR TI=(security) OR TI=(violent) OR TI=(violence) OR TI=(anxiety) OR TI=(anxious) OR TI=(stress) OR TI=(strain) OR TI=(self harm) OR TI=(self injury) OR TI=(suicide) OR TI=(suicidal) OR TI=(sedation) OR TI=(sedate) OR TI=(sedative) OR TI=(restrain) OR TI=(restraint) OR TI=(ligature) OR TI=(risk) OR TI=(resilience) OR TI=(length of stay) OR TI=(leaving without being seen) OR TI=(lwbs) OR TI=(elope) OR TI=(elopement) OR TI=(injure) OR TI=(injury) OR TI=(family engagement) OR TI=(transfer) OR TI=(wellbeing) OR TI=(outpatient follow up) OR TI=(ambulatory follow up) **OR** AB=(defense mechanisms) OR AB=(emotions) OR AB=(behavioral symptoms) OR AB=(length of stay) OR AB=(patient relations) OR AB=(family relations) OR AB=(aggression) OR AB=(aggressive) OR AB=(aggressive) OR AB=(agitated) OR AB=(confusion) OR AB=(distress) OR AB=(anger) OR AB=(angry) OR AB=(comfort) OR AB=(discomfort) OR AB=(pain) OR AB=(satisfaction) OR AB=(dissatisfaction) OR AB=(privacy) OR AB=(private) OR AB=(safe) OR AB=(safety) OR AB=(secure) OR AB=(security) OR AB=(violent) OR AB=(violence) OR AB=(anxiety) OR AB=(anxious) OR AB=(stress) OR AB=(strain) OR AB=(self harm) OR AB=(self injury) OR AB=(suicide) OR AB=(suicidal) OR AB=(sedation) OR AB=(sedate) OR AB=(sedative) OR AB=(restrain) OR AB=(restraint) OR AB=(ligature) OR AB=(risk) OR AB=(resilience) OR AB=(length of stay) OR AB=(leaving without being seen) OR AB=(lwbs) OR AB=(elope) OR AB=(elopement) OR AB=(injure) OR AB=(injury) OR AB=(family engagement) OR AB=(transfer) OR AB=(wellbeing) OR AB=(outpatient follow up) OR AB=(ambulatory follow up))
C) Tools & Technology	Setting	(TI=(emergency service) OR TI=(psychiatric emergency service) OR TI=(emergency department) OR TI=(emergency room) OR TI=(emergency ward) OR TI=(emergency clinic) OR TI=(emergency unit) OR TI=(accident and emergency) **OR** AB=(emergency service) OR AB=(psychiatric emergency service) OR AB=(emergency department) OR AB=(emergency room) OR AB=(emergency ward) OR AB=(emergency clinic) OR AB=(emergency unit) OR AB=(accident and emergency))
	Population	**AND** ((TI=(mental health) OR TI=(behavioral and mental disorders) OR TI=(behavioral health) OR TI=(psychiatry) OR TI=(psychology) OR TI=(psychiatric) OR TI=(autism) OR TI=(autism spectrum) OR TI=(cognitive impairment) OR TI=(intellectual impairment) OR TI=(cognitively impaired) OR TI=(intellectually impaired) OR TI=(depression) OR TI=(anxiety) OR TI=(attention deficit) OR TI=(hyperactivity) OR TI=(adhd) OR TI=(post traumatic stress) OR TI=(ptsd) OR TI=(obsessive compulsive) OR TI=(Tourette) OR TI=(mania) OR TI=(manic) OR TI=(depressive) OR TI=(suicidal) OR TI=(schizophrenia) OR TI=(schizophrenic) OR TI=(eating disorder) OR TI=(anorexia) OR TI=(bulimia) OR TI=(addiction) OR TI=(addicted) OR TI=(overdose) OR TI=(substance abuse) OR TI=(drug abuse) OR TI=(alcohol abuse) OR TI=(intoxicated) OR TI=(intoxication) **OR** AB=(mental health) OR AB=(behavioral and mental disorders) OR AB=(behavioral health) OR AB=(psychiatry) OR AB=(psychology) OR AB=(psychiatric) OR AB=(autism) OR AB=(autism spectrum) OR AB=(cognitive impairment) OR AB=(intellectual impairment) OR AB=(cognitively impaired) OR AB=(intellectually impaired) OR AB=(depression) OR AB=(anxiety) OR AB=(attention deficit) OR AB=(hyperactivity) OR AB=(adhd) OR AB=(post traumatic stress) OR AB=(ptsd) OR AB=(obsessive compulsive) OR AB=(Tourette) OR AB=(mania) OR AB=(manic) OR AB=(depressive) OR AB=(suicidal) OR AB=(schizophrenia) OR AB=(schizophrenic) OR AB=(eating disorder) OR AB=(anorexia) OR AB=(bulimia) OR AB=(addiction) OR AB=(addicted) OR AB=(overdose) OR AB=(substance abuse) OR AB=(drug abuse) OR AB=(alcohol abuse) OR AB=(intoxicated) OR AB=(intoxication)) **AND** (TI=(emergency nursing) OR TI=(emergency medicine) OR TI=(mental health

		personnel) OR TI=(patient) OR TI=(psychiatric patient) OR TI=(family) OR TI=(parent) OR TI =(caregiver) OR TI=(doctor) OR TI=(physician) OR TI=(nurse) OR TI=(clinician) OR TI=(healthcare worker) OR TI=(psychiatrist) OR TI=(psychologist) OR TI=(social worker) OR TI=(counselor) OR TI=(therapist) OR TI=(care team) OR TI=(security) OR TI=(ems) **OR** AB=(emergency nursing) OR AB=(emergency medicine) OR AB=(mental health personnel) OR AB=(patient) OR AB=(family) OR AB=(parent) OR AB=(caregiver) OR AB=(psychiatric patient) OR AB=(doctor) OR AB=(physician) OR AB=(nurse) OR AB=(clinician) OR AB=(healthcare worker) OR AB=(psychiatrist) OR AB=(psychologist) OR AB=(social worker) OR AB=(counselor) OR AB=(therapist) OR AB=(care team) OR AB=(security) OR AB=(ems)))
	Intervention	**AND** (TI=(technology) OR TI=(telemedicine) OR TI=(email) OR TI=(electronic mail) OR TI=(computer) OR TI=(medical informatics) OR TI=(software) OR TI=(telephone) OR TI=(kiosk) OR TI=(mobile app) OR TI=(mobile application) OR TI=(wireless) OR TI=(mobile) OR TI=(text message) OR TI=(message) OR TI=(messaging) OR TI=(sms) OR TI=(short messaging service) OR TI=(mhealth) OR TI=(ehealth) OR TI=(handheld) OR TI=(laptop) OR TI=(palmtop) OR TI=(tablet) OR TI=(smartphone) OR TI=(social network) OR TI=(electronic health record) OR TI=(electronic medical record) OR TI=(ehr) OR TI=(emr) OR TI=(telehealth) OR TI=(telepsychiatry) OR TI=(telepsychology) OR TI=(simulation) **OR** AB=(technology) OR AB=(telemedicine) OR AB=(email) OR AB=(electronic mail) OR AB=(computer) OR AB=(medical informatics) OR AB=(software) OR AB=(telephone) OR AB=(kiosk) OR AB=(mobile app) OR AB=(mobile application) OR AB=(wireless) OR AB=(mobile) OR AB=(text message) OR AB=(message) OR AB=(messaging) OR AB=(sms) OR AB=(short messaging service) OR AB=(mhealth) OR AB=(ehealth) OR AB=(handheld) OR AB=(laptop) OR AB=(palmtop) OR AB=(tablet) OR AB=(smartphone) OR AB=(social network) OR AB=(electronic health record) OR AB=(electronic medical record) OR AB=(ehr) OR AB=(emr) OR AB=(telehealth) OR AB=(telepsychiatry) OR AB=(telepsychology) OR AB=(simulation))
	Outcome	**AND** (TI=(defense mechanisms) OR TI=(emotions) OR TI=(behavioral symptoms) OR TI=(length of stay) OR TI=(patient relations) OR TI=(family relations) OR TI=(aggression) OR TI=(aggressive) OR TI=(aggressive) OR TI=(agitated) OR TI=(confusion) OR TI=(distress) OR TI=(anger) OR TI=(angry) OR TI=(comfort) OR TI=(discomfort) OR TI=(pain) OR TI=(satisfaction) OR TI=(dissatisfaction) OR TI=(privacy) OR TI=(private) OR TI=(safe) OR TI=(safety) OR TI=(secure) OR TI=(security) OR TI=(violent) OR TI=(violence) OR TI=(anxiety) OR TI=(anxious) OR TI=(stress) OR TI=(strain) OR TI=(self harm) OR TI=(self injury) OR TI=(suicide) OR TI=(suicidal) OR TI=(sedation) OR TI=(sedate) OR TI=(sedative) OR TI=(restrain) OR TI=(restraint) OR TI=(ligature) OR TI=(risk) OR TI=(resilience) OR TI=(length of stay) OR TI=(leaving without being seen) OR TI=(lwbs) OR TI=(elope) OR TI=(elopement) OR TI=(injure) OR TI=(injury) OR TI=(family engagement) OR TI=(transfer) OR TI=(wellbeing) OR TI=(outpatient follow up) OR TI=(ambulatory follow up) **OR** AB=(defense mechanisms) OR AB=(emotions) OR AB=(behavioral symptoms) OR AB=(length of stay) OR AB=(patient relations) OR AB=(family relations) OR AB=(aggression) OR AB=(aggressive) OR AB=(aggressive) OR AB=(agitated) OR AB=(confusion) OR AB=(distress) OR AB=(anger) OR AB=(angry) OR AB=(comfort) OR AB=(discomfort) OR AB=(pain) OR AB=(satisfaction) OR AB=(dissatisfaction) OR AB=(privacy) OR AB=(private) OR AB=(safe) OR AB=(safety) OR AB=(secure) OR AB=(security) OR AB=(violent) OR AB=(violence) OR AB=(anxiety) OR AB=(anxious) OR AB=(stress) OR AB=(strain) OR AB=(self harm) OR AB=(self injury) OR AB=(suicide) OR AB=(suicidal) OR AB=(sedation) OR

		AB=(sedate) OR AB=(sedative) OR AB=(restrain) OR AB=(restraint) OR AB=(ligature) OR AB=(risk) OR AB=(resilience) OR AB=(length of stay) OR AB=(leaving without being seen) OR AB=(lwbs) OR AB=(elope) OR AB=(elopement) OR AB=(injure) OR AB=(injury) OR AB=(family engagement) OR AB=(transfer) OR AB=(wellbeing) OR AB=(outpatient follow up) OR AB=(ambulatory follow up))
Engineering Village		
A) Tasks & Organizational Conditions	Setting	(((“emergency service” OR “psychiatric emergency service” OR “emergency department” OR “emergency room” OR ‘emergency ward” OR “emergency clinic” OR “emergency unit” OR “ed” OR “er” OR “accident and emergency”) WN TI) **OR** ((“emergency service” OR “psychiatric emergency service” OR “emergency department” OR “emergency room” OR ‘emergency ward” OR “emergency clinic” OR “emergency unit” OR “accident and emergency”)WN AB))
	Population	**AND** ((((“mental health” OR “behavioral and mental disorders” OR “behavioral health” OR “psychiatry” OR “psychology” OR “psychiatric” OR “autism” OR “autism spectrum” OR “cognitive impairment” OR “intellectual impairment” OR “cognitively impaired” OR “intellectually impaired” OR “depression” OR “anxiety” OR “attention deficit” OR “hyperactivity” OR “adhd” OR “post traumatic stress” OR “ptsd” OR “obsessive compulsive” OR “Tourette” OR “mania” OR “manic” OR “depressive” OR “suicidal” OR “schizophrenia” OR “schizophrenic” OR “eating disorder” OR “anorexia” OR “bulimia” OR “addiction OR “addicted” OR “overdose” OR “substance abuse” OR “drug abuse” OR “alcohol abuse” OR “intoxication” OR “intoxicated”)WN TI) **OR** (“mental health” OR “behavioral and mental disorders” OR “behavioral health” OR “psychiatry” OR “psychology” OR “psychiatric” OR “autism” OR “autism spectrum” OR “cognitive impairment” OR “intellectual impairment” OR “cognitively impaired” OR “intellectually impaired” OR “depression” OR “anxiety” OR “attention deficit” OR “hyperactivity” OR “adhd” OR “post traumatic stress” OR “ptsd” OR “obsessive compulsive” OR “Tourette” OR “mania” OR “manic” OR “depressive” OR “suicidal” OR “schizophrenia” OR “schizophrenic” OR “eating disorder” OR “anorexia” OR “bulimia” OR “addiction OR “addicted” OR “overdose” OR “substance abuse” OR “drug abuse” OR “alcohol abuse” OR “intoxication” OR “intoxicated”)WN AB)) **AND** (((“emergency nursing” OR “emergency medicine” OR “mental health personnel” OR “patient” OR “psychiatric patient” OR “family” OR “parent” OR “caregiver” OR “doctor” OR “physician” OR “nurse” OR “clinician” OR “healthcare worker” OR “psychiatrist” OR “psychologist” OR “social worker” OR “counselor” OR “therapist” OR “care team” OR “security” OR “ems”)WN TI) **OR** ((“emergency nursing” OR “emergency medicine” OR “mental health personnel” OR “patient” OR “psychiatric patient” OR “family” OR “parent” OR “caregiver” OR “doctor” OR “physician” OR “nurse” OR “clinician” OR “healthcare worker” OR “psychiatrist” OR “psychologist” OR “social worker” OR “counselor” OR “therapist” OR “care team” OR “security” OR “ems”)WN AB)))
	Intervention	**AND** (((“continuing education” OR “education” OR “workflow” OR “decision making” OR “task” OR “process” OR “triage” OR “fast track” OR “lean” OR “treatment” OR “assess” OR “assessment” OR “screening” OR “medical screening” OR “psychiatric screening” OR “evaluation” OR “evaluate” OR “training” OR “professional development” OR “mentor” OR “mentorship” OR “learning” OR “culture” OR “management” OR “manager” OR “change” OR “leader” OR “leadership” OR “team” OR “teamwork” OR “policy” OR “policies” OR “staffing” OR “scheduling” OR “schedule”)WN TI) **OR** ((“continuing education” OR “education” OR “workflow” OR “decision making” OR “task” OR “process” OR “triage” OR “fast track” OR “lean” OR “treatment” OR “assess” OR “assessment” OR “screening” OR “medical screening” OR

		"psychiatric screening" OR "evaluation" OR "evaluate" OR "training" OR "professional development" OR "mentor" OR "mentorship" OR "learning" OR "culture" OR "management" OR "manager" OR "change" OR "leader" OR "leadership" OR "team" OR "teamwork" OR "policy" OR "policies" OR "staffing" OR "scheduling" OR "schedule")WN AB))
	Outcome	**AND** ((("defense mechanisms" OR "emotions" OR "behavioral symptoms" OR "length of stay" OR "patient relations" OR "family relations" OR "aggression" OR "aggressive" OR "aggressive" OR "agitated" OR "confusion" OR "distress" OR "anger" OR "angry" OR "comfort" OR "discomfort" OR "pain" OR "satisfaction" OR "dissatisfaction" OR "privacy" OR "private" OR "safe" OR "safety" OR "secure" OR "security" OR "violent" OR "violence" OR "anxiety" OR "anxious" OR "stress" OR "strain" OR "self harm" OR "self injury" OR "suicide" OR "suicidal" OR "sedation" OR "sedate" OR "sedative" OR "restrain" OR "restraint" OR "ligature" OR "risk" OR "resilience" OR "length of stay" OR "leaving without being seen" OR "lwbs" OR "elope" OR "elopement" OR "injure" OR "injury" OR "family engagement" OR "transfer" OR "wellbeing" OR "outpatient follow up" OR "ambulatory follow up") WN TI) **OR** (("defense mechanisms" OR "emotions" OR "behavioral symptoms" OR "length of stay" OR "patient relations" OR "family relations" OR "aggression" OR "aggressive" OR "aggressive" OR "agitated" OR "confusion" OR "distress" OR "anger" OR "angry" OR "comfort" OR "discomfort" OR "pain" OR "satisfaction" OR "dissatisfaction" OR "privacy" OR "private" OR "safe" OR "safety" OR "secure" OR "security" OR "violent" OR "violence" OR "anxiety" OR "anxious" OR "stress" OR "strain" OR "self harm" OR "self injury" OR "suicide" OR "suicidal" OR "sedation" OR "sedate" OR "sedative" OR "restrain" OR "restraint" OR "ligature" OR "risk" OR "resilience" OR "length of stay" OR "leaving without being seen" OR "lwbs" OR "elope" OR "elopement" OR "injure" OR "injury" OR "family engagement" OR "transfer" OR "wellbeing" OR "outpatient follow up" OR "ambulatory follow up") WN AB))
B) Built Environment	Setting	((("emergency service" OR "psychiatric emergency service" OR "emergency department" OR "emergency room" OR 'emergency ward" OR "emergency clinic" OR "emergency unit" OR "ed" OR "er" OR "accident and emergency") WN TI) **OR** (("emergency service" OR "psychiatric emergency service" OR "emergency department" OR "emergency room" OR 'emergency ward" OR "emergency clinic" OR "emergency unit" OR "accident and emergency")WN AB))
	Population	**AND** (((("mental health" OR "behavioral and mental disorders" OR "behavioral health" OR "psychiatry" OR "psychology" OR "psychiatric" OR "autism" OR "autism spectrum" OR "cognitive impairment" OR "intellectual impairment" OR "cognitively impaired" OR "intellectually impaired" OR "depression" OR "anxiety" OR "attention deficit" OR "hyperactivity" OR "adhd" OR "post traumatic stress" OR "ptsd" OR "obsessive compulsive" OR "Tourette" OR "mania" OR "manic" OR "depressive" OR "suicidal" OR "schizophrenia" OR "schizophrenic" OR "eating disorder" OR "anorexia" OR "bulimia" OR "addiction OR "addicted" OR "overdose" OR "substance abuse" OR "drug abuse" OR "alcohol abuse" OR "intoxication" OR "intoxicated")WN TI) **OR** ("mental health" OR "behavioral and mental disorders" OR "behavioral health" OR "psychiatry" OR "psychology" OR "psychiatric" OR "autism" OR "autism spectrum" OR "cognitive impairment" OR "intellectual impairment" OR "cognitively impaired" OR "intellectually impaired" OR "depression" OR "anxiety" OR "attention deficit" OR "hyperactivity" OR "adhd" OR "post traumatic stress" OR "ptsd" OR "obsessive compulsive" OR "Tourette" OR "mania" OR "manic" OR "depressive" OR "suicidal" OR "schizophrenia" OR "schizophrenic" OR "eating disorder" OR "anorexia" OR "bulimia" OR "addiction OR "addicted" OR "overdose" OR "substance abuse" OR "drug abuse" OR "alcohol abuse" OR "intoxication" OR "intoxicated")WN AB)) **AND**

		(((“emergency nursing” OR “emergency medicine” OR “mental health personnel” OR “patient” OR “psychiatric patient” OR “family” OR “parent” OR “caregiver” OR “doctor” OR “physician” OR “nurse” OR “clinician” OR “healthcare worker” OR “psychiatrist” OR “psychologist” OR “social worker” OR “counselor” OR “therapist” OR “care team” OR “security” OR “ems”)WN TI) **OR** ((“emergency nursing” OR “emergency medicine” OR “mental health personnel” OR “patient” OR “psychiatric patient” OR “family” OR “parent” OR “caregiver” OR “doctor” OR “physician” OR “nurse” OR “clinician” OR “healthcare worker” OR “psychiatrist” OR “psychologist” OR “social worker” OR “counselor” OR “therapist” OR “care team” OR “security” OR “ems”)WN AB)))
	Intervention	**AND** (((“built environment” OR “hospital design” OR “construction” OR “built environment” OR “physical environment” OR “architecture” OR “interior design” OR “lighting” OR “daylight” OR “window” OR “crisis stabilization” OR “layout” OR “visibility” OR “furniture” OR “decoration” OR “décor” OR “art” OR “positive distraction” OR “noise” OR “ergonomics” OR “ergonomic” OR “odor” OR “smell” OR “anti ligature” OR “ligature resistant” OR “sensory room” OR “Snoezelen”) WN TI) **OR** ((“built environment” OR “hospital design” OR “construction” OR “built environment” OR “physical environment” OR “architecture” OR “interior design” OR “lighting” OR “daylight” OR “window” OR “crisis stabilization” OR “layout” OR “visibility” OR “furniture” OR “decoration” OR “décor” OR “art” OR “positive distraction” OR “noise” OR “ergonomics” OR “ergonomic” OR “odor” OR “smell” OR “anti ligature” OR “ligature resistant” OR “sensory room” OR “Snoezelen”) WN TI))
	Outcome	**AND** (((“defense mechanisms” OR “emotions” OR “behavioral symptoms” OR “length of stay” OR “patient relations” OR “family relations” OR “aggression” OR “aggressive” OR “aggressive” OR “agitated” OR “confusion” OR “distress” OR “anger” OR “angry” OR “comfort” OR “discomfort” OR “pain” OR “satisfaction” OR “dissatisfaction” OR “privacy” OR “private” OR “safe” OR “safety” OR “secure” OR “security” OR “violent” OR “violence” OR “anxiety” OR “anxious” OR “stress” OR “strain” OR “self harm” OR “self injury” OR “suicide” OR “suicidal” OR “sedation” OR “sedate” OR “sedative” OR “restrain” OR “restraint” OR “ligature” OR “risk” OR “resilience” OR “length of stay” OR “leaving without being seen” OR “lwbs” OR “elope” OR “elopement” OR “injure” OR “injury” OR “family engagement” OR “transfer” OR “wellbeing” OR “outpatient follow up” OR “ambulatory follow up”) WN TI) **OR** ((“defense mechanisms” OR “emotions” OR “behavioral symptoms” OR “length of stay” OR “patient relations” OR “family relations” OR “aggression” OR “aggressive” OR “aggressive” OR “agitated” OR “confusion” OR “distress” OR “anger” OR “angry” OR “comfort” OR “discomfort” OR “pain” OR “satisfaction” OR “dissatisfaction” OR “privacy” OR “private” OR “safe” OR “safety” OR “secure” OR “security” OR “violent” OR “violence” OR “anxiety” OR “anxious” OR “stress” OR “strain” OR “self harm” OR “self injury” OR “suicide” OR “suicidal” OR “sedation” OR “sedate” OR “sedative” OR “restrain” OR “restraint” OR “ligature” OR “risk” OR “resilience” OR “length of stay” OR “leaving without being seen” OR “lwbs” OR “elope” OR “elopement” OR “injure” OR “injury” OR “family engagement” OR “transfer” OR “wellbeing” OR “outpatient follow up” OR “ambulatory follow up”) WN AB))
C) Tools & Technology	Setting	(((“emergency service” OR “psychiatric emergency service” OR “emergency department” OR “emergency room” OR ‘emergency ward” OR “emergency clinic” OR “emergency unit” OR “ed” OR “er” OR “accident and emergency”) WN TI) **OR** ((“emergency service” OR “psychiatric emergency service” OR “emergency department” OR “emergency room” OR ‘emergency ward” OR “emergency clinic” OR “emergency unit” OR “accident and emergency”)WN AB))

	Population	**AND** ((((“mental health” OR “behavioral and mental disorders” OR “behavioral health” OR “psychiatry” OR “psychology” OR “psychiatric” OR “autism” OR “autism spectrum” OR “cognitive impairment” OR “intellectual impairment” OR “cognitively impaired” OR “intellectually impaired” OR “depression” OR “anxiety” OR “attention deficit” OR “hyperactivity” OR “adhd” OR “post traumatic stress” OR “ptsd” OR “obsessive compulsive” OR “Tourette” OR “mania” OR “manic” OR “depressive” OR “suicidal” OR “schizophrenia” OR “schizophrenic” OR “eating disorder” OR “anorexia” OR “bulimia” OR “addiction OR “addicted” OR “overdose” OR “substance abuse” OR “drug abuse” OR “alcohol abuse” OR “intoxication” OR “intoxicated”)WN TI) **OR** (“mental health” OR “behavioral and mental disorders” OR “behavioral health” OR “psychiatry” OR “psychology” OR “psychiatric” OR “autism” OR “autism spectrum” OR “cognitive impairment” OR “intellectual impairment” OR “cognitively impaired” OR “intellectually impaired” OR “depression” OR “anxiety” OR “attention deficit” OR “hyperactivity” OR “adhd” OR “post traumatic stress” OR “ptsd” OR “obsessive compulsive” OR “Tourette” OR “mania” OR “manic” OR “depressive” OR “suicidal” OR “schizophrenia” OR “schizophrenic” OR “eating disorder” OR “anorexia” OR “bulimia” OR “addiction OR “addicted” OR “overdose” OR “substance abuse” OR “drug abuse” OR “alcohol abuse” OR “intoxication” OR “intoxicated”)WN AB)) **AND** (((“emergency nursing” OR “emergency medicine” OR “mental health personnel” OR “patient” OR “psychiatric patient” OR “family” OR “parent” OR “caregiver” OR “doctor” OR “physician” OR “nurse” OR “clinician” OR “healthcare worker” OR “psychiatrist” OR “psychologist” OR “social worker” OR “counselor” OR “therapist” OR “care team” OR “security” OR “ems”)WN TI) **OR** ((“emergency nursing” OR “emergency medicine” OR “mental health personnel” OR “patient” OR “psychiatric patient” OR “family” OR “parent” OR “caregiver” OR “doctor” OR “physician” OR “nurse” OR “clinician” OR “healthcare worker” OR “psychiatrist” OR “psychologist” OR “social worker” OR “counselor” OR “therapist” OR “care team” OR “security” OR “ems”)WN AB)))
	Intervention	**AND** (((“technology” OR “telemedicine” OR “email” OR “electronic mail” OR “computer” OR “medical informatics” OR “software” OR “telephone” OR “kiosk” OR “mobile app” OR “mobile application” OR “wireless” OR “mobile” OR “text message” OR “message” OR “messaging” OR “sms” OR “short messaging service” OR “mhealth” OR “ehealth” OR “handheld” OR “laptop” OR “palmtop” OR “tablet” OR “smartphone” OR “social network” OR “electronic health record” OR “electronic medical record” OR “ehr" OR “emr” OR “telehealth” OR “telepsychiatry” OR “telepsychology” OR “simulation”) WN TI) **OR** ((“technology” OR “telemedicine” OR “email” OR “electronic mail” OR “computer” OR “medical informatics” OR “software” OR “telephone” OR “kiosk” OR “mobile app” OR “mobile application” OR “wireless” OR “mobile” OR “text message” OR “message” OR “messaging” OR “sms” OR “short messaging service” OR “mhealth” OR “ehealth” OR “handheld” OR “laptop” OR “palmtop” OR “tablet” OR “smartphone” OR “social network” OR “electronic health record” OR “electronic medical record” OR “ehr" OR “emr” OR “telehealth” OR “telepsychiatry” OR “telepsychology” OR “simulation”) WN TI))
	Outcome	**AND** (((“defense mechanisms” OR “emotions” OR “behavioral symptoms” OR “length of stay” OR “patient relations” OR “family relations” OR “aggression” OR “aggressive” OR “aggressive” OR “agitated” OR “confusion” OR “distress” OR “anger” OR “angry” OR “comfort” OR “discomfort” OR “pain” OR “satisfaction” OR “dissatisfaction” OR “privacy” OR “private” OR “safe” OR “safety” OR “secure” OR “security” OR “violent” OR “violence” OR “anxiety” OR “anxious” OR “stress” OR “strain” OR “self harm” OR “self injury” OR “suicide” OR “suicidal” OR “sedation” OR “sedate” OR “sedative” OR

		"restrain" OR "restraint" OR "ligature" OR "risk" OR "resilience" OR "length of stay" OR "leaving without being seen" OR "lwbs" OR "elope" OR "elopement" OR "injure" OR "injury" OR "family engagement" OR "transfer" OR "wellbeing" OR "outpatient follow up" OR "ambulatory follow up") WN TI) **OR** (("defense mechanisms" OR "emotions" OR "behavioral symptoms" OR "length of stay" OR "patient relations" OR "family relations" OR "aggression" OR "aggressive" OR "aggressive" OR "agitated" OR "confusion" OR "distress" OR "anger" OR "angry" OR "comfort" OR "discomfort" OR "pain" OR "satisfaction" OR "dissatisfaction" OR "privacy" OR "private" OR "safe" OR "safety" OR "secure" OR "security" OR "violent" OR "violence" OR "anxiety" OR "anxious" OR "stress" OR "strain" OR "self harm" OR "self injury" OR "suicide" OR "suicidal" OR "sedation" OR "sedate" OR "sedative" OR "restrain" OR "restraint" OR "ligature" OR "risk" OR "resilience" OR "length of stay" OR "leaving without being seen" OR "lwbs" OR "elope" OR "elopement" OR "injure" OR "injury" OR "family engagement" OR "transfer" OR "wellbeing" OR "outpatient follow up" OR "ambulatory follow up") WN AB))
